# Supplementary material for: ACE inhibition and cardiometabolic risk factors, lung ACE2 and TMPRSS2 gene expression, and plasma ACE2 levels: a Mendelian randomization study
Source: R Soc Open Sci. 2020 Nov 18;7(11):200958. doi: 10.1098/rsos.200958 (PMC7735342; doi:10.1098/rsos.200958)
Supplement: Supplementary Tables and Figures [file rsos200958supp1.docx]

**Supplement**

**ACE inhibition and cardiometabolic risk factors, lung *ACE2* and *TMPRSS2* gene expression, and plasma ACE2 levels: a Mendelian randomization study**

Dipender Gill^1^* BMBCh PhD, Marios Arvanitis^2^ MD, Paul Carter^3^ MBBS, Ana I. Hernández Cordero^4^ PhD, Brian Jo^5^ BS, Ville Karhunen^1^ MSc, Susanna C. Larsson^6,7^ PhD, Xuan Li^4^ MSc, Sam M. Lockhart^8^ MBBCh, Amy Mason^9,10^ PhD, Evanthia Pashos^11^ PhD, Ashis Saha^12^ MS, Vanessa Y. Tan^13,14^ PhD, Verena Zuber^1,15^ PhD, Yohan Bossé^16^ PhD, Sarah Fahle^9,10,17^ PhD, Ke Hao^18^ ScD, Tao Jiang^9^ PhD, Philippe Joubert^16^ MD PhD, Alan C. Lunt^1^ PhD, Willem Hendrik Ouwehand^20,21^ PhD, David J. Roberts^17,22,23^ MBChB PhD, Wim Timens^24^ MD PhD, Maarten van den Berge^25^ MD PhD, Nicholas A. Watkins^17,26^ PhD, Alexis Battle^27^ PhD, Adam S. Butterworth^9,10,17,28,29^ PhD, John Danesh^9,10,17,21,28,29^ FMedSci, Emanuele Di Angelantonio^9,10,17,21,28,29^ FRCP, Barbara E. Engelhardt^30^ PhD, James E. Peters^29,31^ MBChB PhD, Don D. Sin^4^ MD, Stephen Burgess^9,10,15,28,32^† PhD

**Address for correspondence**

*Dr Dipender Gill

Department of Epidemiology and Biostatistics, Medical School Building, St Mary's Hospital, Imperial College London, United Kingdom, W2 1PG

Telephone: +44 (0) 7904843810

Email: [dipender.gill@imperial.ac.uk](mailto:dipender.gill@imperial.ac.uk)

†Dr Stephen Burgess

MRC Biostatistics Unit, Cambridge Institute of Public Health, Robinson Way, Cambridge, United Kingdom, CB2 0SR

Telephone: +44 (0) 7759988359

Email: [sb452@medschl.cam.ac.uk](mailto:sb452@medschl.cam.ac.uk)

**Contents**

[**Supplementary Figure 1: Genetic associations with serum ACE level (horizontal axis, standard deviation units) plotted against a) systolic blood pressure in the UK Biobank (Carter et al. BMJ. 2019;365:l1855), b) genetic associations with *ACE2* gene expression in lung from the GTEx project, c) genetic associations with *TMPRSS2* gene expression in lung from the GTEx project, d) genetic associations with *ACE2* gene expression in lung from the Lung eQTL consortium, e and f) genetic associations with *TMPRSS2* gene expression in lung from the Lung eQTL consortium (probes 1 and 2), and g) genetic associations with plasma ACE2 concentrations in INTERVAL.** 3](#_Toc53913548)

[**Supplementary Figure 2: Genetic associations with *ACE2* and *TMPRSS2* gene expression in the lung (GTEx and Lung consortium) and circulating ACE2 protein levels in the plasma (INTERVAL) a) per one standard deviation increased ACE concentration conferred through 12 genome-wide significant variants at the ACE gene, and b) per blood pressure-lowering allele for the rs4343 variant in the ACE gene (bottom panel; results not available for the GTEx consortium because the rs4343 variant is missing). The two sets of results for *TMPRSS2* expression in the Lung eQTL consortium refer to two separate probe sets for estimating gene expression.** 4](#_Toc53913549)

[**Supplementary Figure 3: Genetic associations with type 2 diabetes mellitus (horizontal axis, log odds ratios) for up to 202 variants associated with type 2 diabetes mellitus at a genome-wide level of significance plotted against a) genetic associations with *ACE2* gene expression in lung from the GTEx project, b) genetic associations with *ACE2* gene expression in lung from the Lung eQTL consortium, and c) genetic associations with plasma ACE2 concentrations in INTERVAL.** 5](#_Toc53913550)

[**Supplementary Table 1. The specific probes for the two *TMPRSS2* probe sets.** 6](#_Toc53913551)

[**Supplementary Table 2. Genetic variants at the *ACE* locus related to serum ACE levels that were used as genetic proxies for ACEi inhibitor drug effects.** 7](#_Toc53913552)

[**Supplementary Table 3. Genetic variant at the *ACE* locus related to systolic blood pressure that was used as a genetic proxy for ACEi inhibitor drug effects.** 8](#_Toc53913553)

[**Supplementary Table 4. Genetic variants that were used as proxies for body mass index (after clumping).** 9](#_Toc53913554)

[**Supplementary Table 5. Genetic variants that were used as proxies for liability to chronic obstructive pulmonary disease (after clumping).** 22](#_Toc53913555)

[**Supplementary Table 6. Genetic variants that were used as proxies for lifetime smoking index (after clumping).** 24](#_Toc53913556)

[**Supplementary Table 7. Genetic variants that were used as proxies for low-density lipoprotein cholesterol (after clumping).** 27](#_Toc53913557)

[**Supplementary Table 8. Genetic variants that were used as proxies for systolic blood pressure (after clumping).** 29](#_Toc53913558)

[**Supplementary Table 9. Genetic variants that were used as proxies for liability to type 2 diabetes mellitus (after clumping).** 34](#_Toc53913559)

[**Supplementary Table 10: Pleiotropy robust Mendelian randomization method estimates for analyses investigating the association of genetic liability to type 2 diabetes with lung *ACE2* expression in the GTEx project and Lung eQTL consortium and with plasma concentrations of ACE2 in INTERVAL.** 39](#_Toc53913560)

# **Supplementary Figure 1: Genetic associations with serum ACE level (horizontal axis, standard deviation units) plotted against a) systolic blood pressure in the UK Biobank (Carter et al. BMJ. 2019;365:l1855), b) genetic associations with *ACE2* gene expression in lung from the GTEx project, c) genetic associations with *TMPRSS2* gene expression in lung from the GTEx project, d) genetic associations with *ACE2* gene expression in lung from the Lung eQTL consortium, e and f) genetic associations with *TMPRSS2* gene expression in lung from the Lung eQTL consortium (probes 1 and 2), and g) genetic associations with plasma ACE2 concentrations in INTERVAL.**

a.
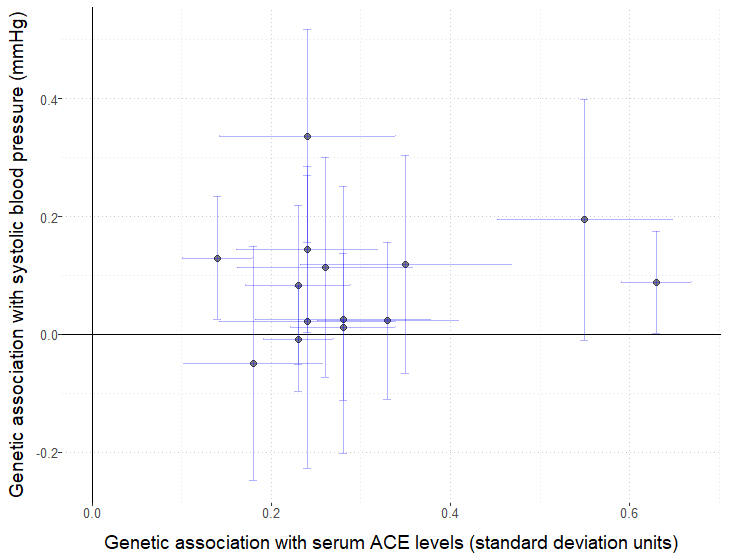
 b.
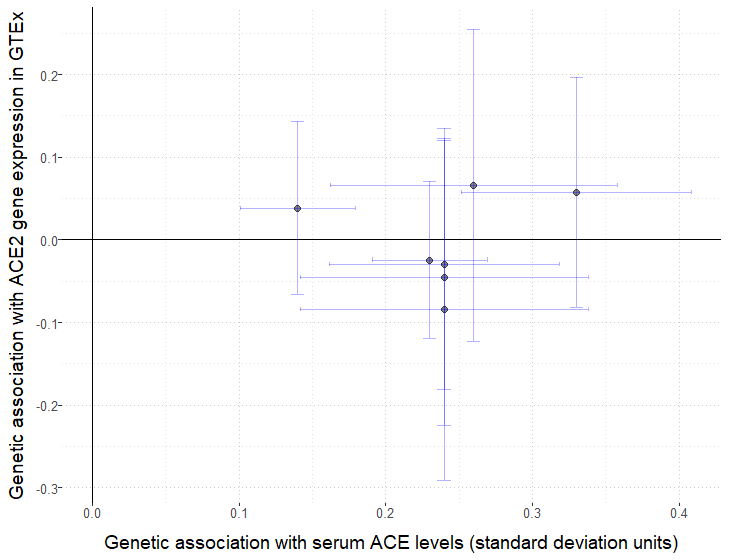


c.
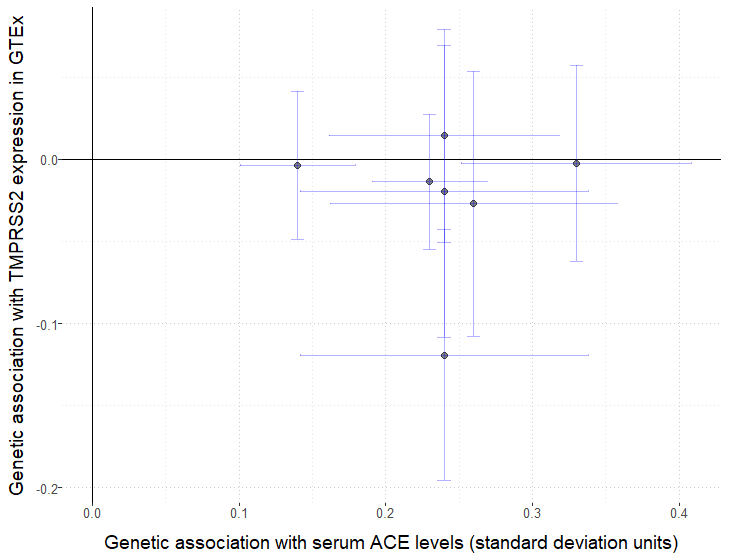
 d.
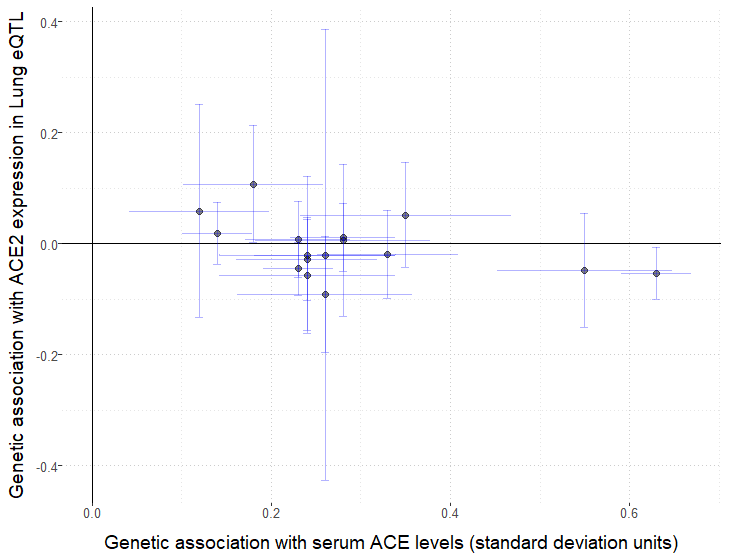


e.
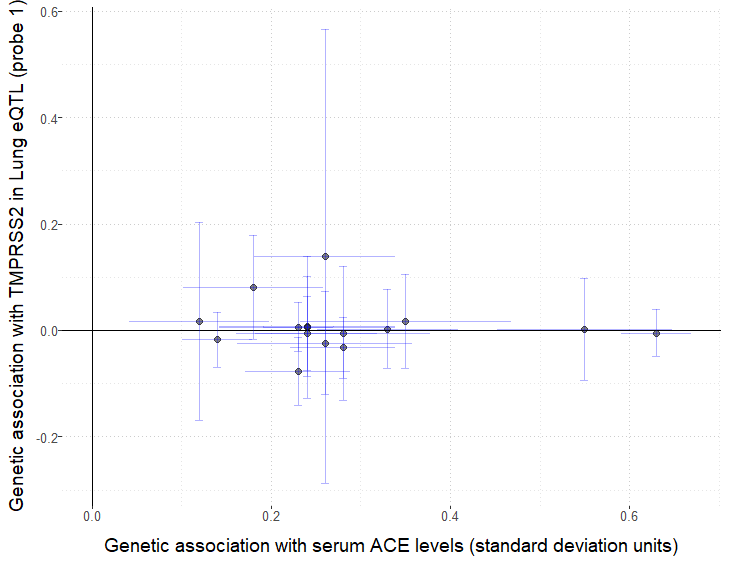
 f.
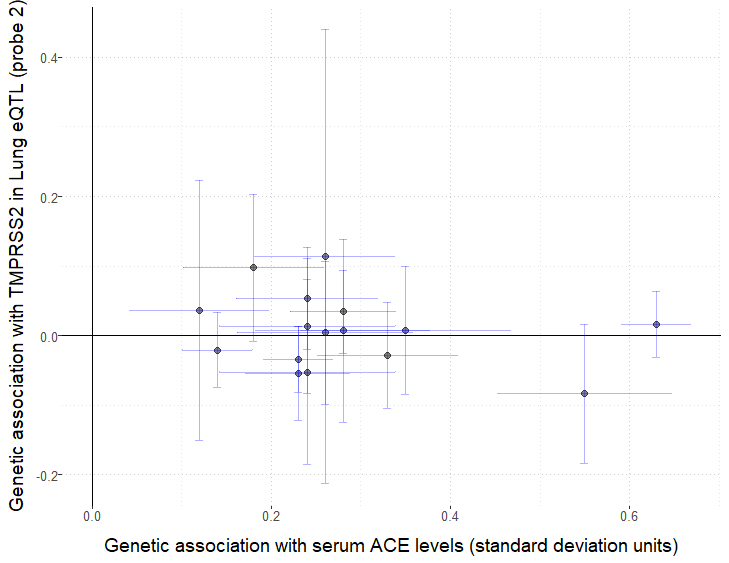


g.
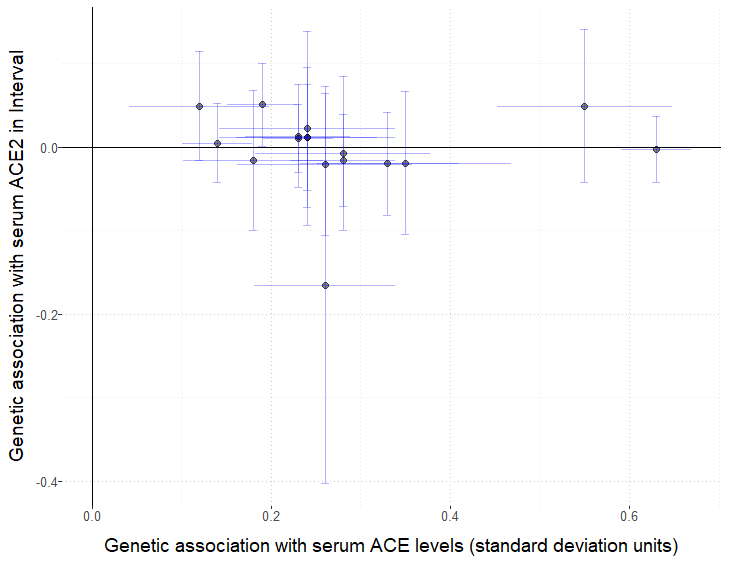


# **Supplementary Figure 2: Genetic associations with *ACE2* and *TMPRSS2* gene expression in the lung (GTEx and Lung consortium) and circulating ACE2 protein levels in the plasma (INTERVAL) a) per one standard deviation increased ACE concentration conferred through 12 genome-wide significant variants at the ACE gene, and b) per blood pressure-lowering allele for the rs4343 variant in the ACE gene (bottom panel; results not available for the GTEx consortium because the rs4343 variant is missing). The two sets of results for *TMPRSS2* expression in the Lung eQTL consortium refer to two separate probe sets for estimating gene expression.**

a.


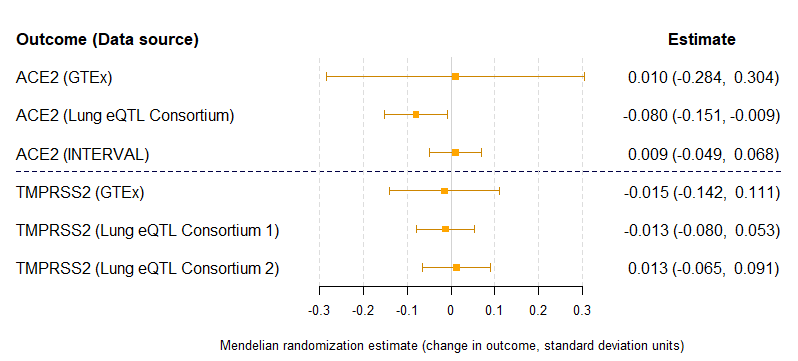


b.


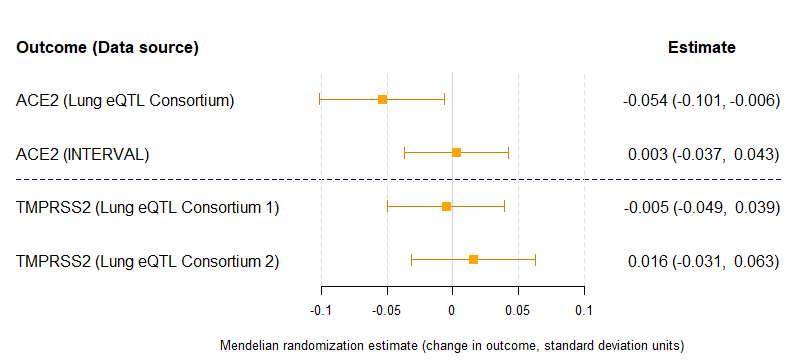


# **Supplementary Figure 3: Genetic associations with type 2 diabetes mellitus (horizontal axis, log odds ratios) for up to 202 variants associated with type 2 diabetes mellitus at a genome-wide level of significance plotted against a) genetic associations with *ACE2* gene expression in lung from the GTEx project, b) genetic associations with *ACE2* gene expression in lung from the Lung eQTL consortium, and c) genetic associations with plasma ACE2 concentrations in INTERVAL.**

Genetic associations with the outcome measures are in standard deviation units.


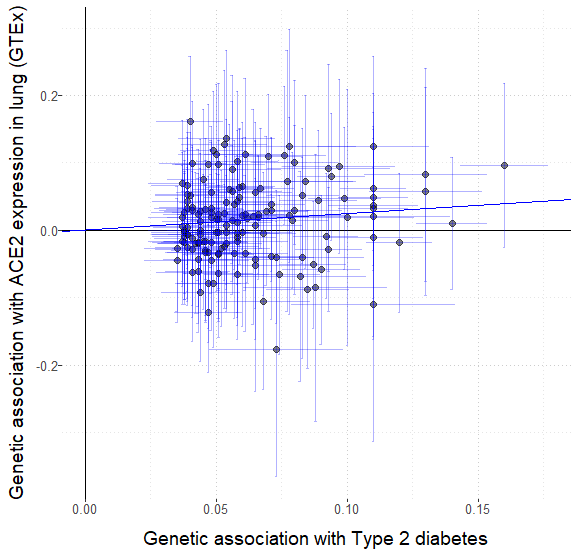
a)


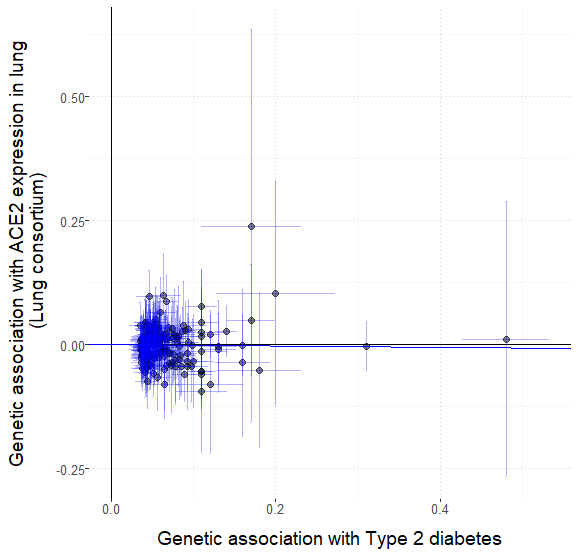
b)


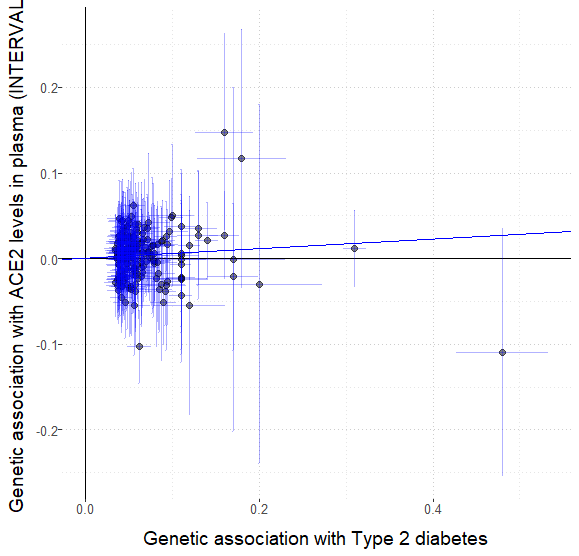
c)

# **Supplementary Table 1. The specific probes for the two *TMPRSS2* probe sets.**

| **Probe set** | **Probe sequence** | **Binding** |
| --- | --- | --- |
| Probe set 1 (100130004_TGI_at) | GTTTTGTTTTGGACTCTCTGTGGTC | Exon 14 TMPRSS2 |
|  | TTTGTTTTGGACTCTCTGTGGTCCC | Exon 14 TMPRSS2 |
|  | GCTTTGACAAAATGACTGGCTCCTG | Exon 14 TMPRSS2 |
|  | TTGCCAAGTAAGAGTGGTGGCCTAT | Exon 14 TMPRSS2 |
|  | TGCCAAGTAAGAGTGGTGGCCTATT | Exon 14 TMPRSS2 |
|  | TTTGACAAAATGACTGGCTCCTGAC | Exon 14 TMPRSS2 |
|  | TCACCTTTGCCAAGTAAGAGTGGTG | Exon 14 TMPRSS2 |
|  | TTTTGTTTTGGACTCTCTGTGGTCC | Exon 14 TMPRSS2 |
| Probe set 2 (100157336_TGI_at) | GTCCCCTGCTACAGGGCATTGAGGT | Exon 2 AK313338 |
|  | CCACCTCCCCACTTAGAGAATATTT | intronic |
|  | AGTTTCGTAACTCCTGCCGCATAGT | intronic |
|  | CAGAGCTTTGAGAAGGCTGTTATCA | intronic |
|  | ATTGAGGTGAGGTCCGCCTTTGCCC | intronic |
|  | TGTTTCTTTTTGAACTTGCCACCTC | intronic |
|  | GAGAGCGCTGCTTTCAGAGCTTTGA | intronic |
|  | AGAGAGTGACCCGTGCATCTTTCCA | Exon 2 AK313338 |
|  | TGCCGCATAGTTGGTGCCTGCTCTC | intronic |

#

# **Supplementary Table 2. Genetic variants at the *ACE* locus related to serum ACE levels that were used as genetic proxies for ACEi inhibitor drug effects.**

| Variant | Effect Allele | Beta | Standard error | P |
| --- | --- | --- | --- | --- |
| rs4343 | A | -0.63 | 0.02 | 1.53E-213 |
| rs1074637 | T | -0.24 | 0.04 | 4.40E-09 |
| rs11650201 | G | -0.28 | 0.03 | 2.69E-18 |
| rs117808108 | T | -0.12 | 0.04 | 1.13E-03 |
| rs12452187 | A | -0.23 | 0.02 | 2.53E-27 |
| rs12602457 | G | -0.23 | 0.03 | 2.64E-14 |
| rs13342595 | C | -0.14 | 0.02 | 2.48E-09 |
| rs1443431 | A | -0.24 | 0.05 | 4.20E-06 |
| rs2137143 | T | -0.35 | 0.06 | 7.50E-09 |
| rs4313 | T | -0.24 | 0.05 | 1.53E-05 |
| rs4968780 | C | -0.28 | 0.05 | 1.86E-08 |
| rs71377703 | C | -0.18 | 0.04 | 1.07E-06 |
| rs72845888 | T | -0.26 | 0.05 | 1.50E-07 |
| rs72847305 | A | -0.33 | 0.04 | 4.52E-17 |
| rs74251225 | G | -0.26 | 0.04 | 1.57E-10 |
| rs75457471 | A | -0.19 | 0.02 | 8.10E-15 |
| rs79480822 | C | -0.55 | 0.05 | 6.37E-24 |

# **Supplementary Table 3. Genetic variant at the *ACE* locus related to systolic blood pressure that was used as a genetic proxy for ACEi inhibitor drug effects.**

| Variant | Effect Allele | Effect | Standard Error | P |
| --- | --- | --- | --- | --- |
| rs4291 | A | -0.2839 | 0.0312 | 8.65E-20 |

# **Supplementary Table 4. Genetic variants that were used as proxies for body mass index (after clumping).**

| Variant | Effect Allele | Effect | Standard Error | P |
| --- | --- | --- | --- | --- |
| rs79113395 | A | -0.02 | 0.0022 | 2.03E-20 |
| rs10909880 | T | -0.0135 | 0.0016 | 2.21E-16 |
| rs11121210 | T | -0.0111 | 0.0017 | 2.37E-10 |
| rs10779751 | A | 0.0131 | 0.0018 | 2.66E-13 |
| rs10803390 | A | -0.0102 | 0.0018 | 1.86E-08 |
| rs61740466 | A | -0.0142 | 0.0022 | 2.24E-10 |
| rs4655141 | T | -0.0173 | 0.0022 | 6.20E-15 |
| rs2228550 | T | -0.0124 | 0.002 | 5.72E-10 |
| rs4653017 | T | 0.0118 | 0.0018 | 1.11E-10 |
| rs7512146 | T | -0.0097 | 0.0017 | 1.29E-08 |
| rs11577094 | T | 0.0186 | 0.003 | 3.28E-10 |
| rs112646560 | T | 0.018 | 0.0023 | 1.29E-14 |
| rs2984618 | T | 0.0165 | 0.0016 | 3.80E-24 |
| rs657452 | A | 0.0188 | 0.0016 | 3.17E-30 |
| rs587271 | T | 0.012 | 0.0018 | 6.21E-11 |
| rs79906980 | T | 0.0158 | 0.0026 | 1.69E-09 |
| rs12140153 | T | -0.0353 | 0.0034 | 1.44E-25 |
| rs2503185 | A | 0.013 | 0.0017 | 1.33E-14 |
| rs3101336 | T | -0.0254 | 0.0016 | 4.80E-54 |
| rs6656785 | A | -0.0178 | 0.0016 | 3.06E-27 |
| rs34517439 | A | 0.0391 | 0.003 | 3.40E-39 |
| rs6696828 | C | 0.012 | 0.0018 | 6.42E-11 |
| rs321237 | A | 0.0126 | 0.002 | 1.49E-10 |
| rs11165643 | T | 0.0185 | 0.0016 | 4.49E-30 |
| rs12072739 | A | -0.0169 | 0.0023 | 1.50E-13 |
| rs17024393 | T | -0.0644 | 0.0049 | 7.11E-39 |
| rs197374 | T | 0.0141 | 0.0017 | 3.16E-16 |
| rs7534091 | A | -0.012 | 0.0018 | 6.65E-11 |
| rs74887628 | A | 0.0305 | 0.0054 | 1.76E-08 |
| rs4970991 | T | 0.0116 | 0.0021 | 1.81E-08 |
| rs61813324 | T | 0.0289 | 0.0028 | 3.20E-24 |
| rs1750307 | A | 0.0129 | 0.0018 | 2.78E-13 |
| rs10733051 | A | 0.0093 | 0.0016 | 6.96E-09 |
| rs61828641 | A | 0.0223 | 0.003 | 2.49E-13 |
| rs543874 | A | -0.0479 | 0.002 | 3.06E-125 |
| rs10920678 | A | 0.0149 | 0.0016 | 7.15E-20 |
| rs2400414 | T | -0.0126 | 0.0018 | 5.52E-13 |
| rs2820295 | A | 0.0235 | 0.0018 | 5.56E-39 |
| rs10920336 | A | -0.0101 | 0.0017 | 4.20E-09 |
| rs1006317 | T | 0.016 | 0.0026 | 3.75E-10 |
| rs6661316 | T | 0.012 | 0.0016 | 1.72E-13 |
| rs10864728 | A | 0.011 | 0.0019 | 1.20E-08 |
| rs946824 | T | 0.0197 | 0.0025 | 4.82E-15 |
| rs6710091 | C | 0.0103 | 0.0018 | 5.78E-09 |
| rs77165542 | T | -0.0939 | 0.0053 | 1.51E-70 |
| rs13021737 | A | -0.0578 | 0.0021 | 2.89E-161 |
| rs10929925 | A | -0.0142 | 0.0016 | 3.05E-18 |
| rs11902450 | T | 0.0167 | 0.0027 | 1.06E-09 |
| rs10182181 | A | -0.0327 | 0.0016 | 2.45E-91 |
| rs935166 | A | -0.0152 | 0.0019 | 8.69E-16 |
| rs10168563 | A | 0.0126 | 0.0018 | 9.33E-12 |
| rs3770890 | T | -0.0297 | 0.0053 | 2.10E-08 |
| rs10185199 | A | -0.0143 | 0.002 | 3.18E-13 |
| rs10169594 | T | -0.012 | 0.0018 | 1.34E-11 |
| rs7561278 | T | 0.0169 | 0.0021 | 4.89E-16 |
| rs930295 | A | 0.0208 | 0.0023 | 2.03E-19 |
| rs805412 | A | -0.0098 | 0.0017 | 1.14E-08 |
| rs4671328 | T | 0.0214 | 0.0017 | 3.42E-36 |
| rs6545714 | A | -0.0194 | 0.0016 | 4.01E-32 |
| rs2861685 | T | 0.0165 | 0.0019 | 7.82E-18 |
| rs10520330 | C | -0.0144 | 0.0024 | 3.57E-09 |
| rs12714199 | T | -0.0141 | 0.0017 | 3.22E-16 |
| rs4303732 | T | 0.0169 | 0.0017 | 1.26E-22 |
| rs264941 | A | -0.0124 | 0.0017 | 1.84E-13 |
| rs10197031 | T | -0.0161 | 0.0019 | 5.05E-18 |
| rs13033310 | A | 0.0146 | 0.0022 | 3.40E-11 |
| rs4988235 | A | 0.0124 | 0.0017 | 7.09E-13 |
| rs17814208 | A | -0.0129 | 0.002 | 7.24E-11 |
| rs6430068 | A | 0.0155 | 0.0026 | 4.45E-09 |
| rs1451077 | A | -0.0169 | 0.0019 | 1.43E-18 |
| rs62176993 | A | 0.0119 | 0.0019 | 8.63E-10 |
| rs3764835 | A | -0.013 | 0.0024 | 3.84E-08 |
| rs12692596 | T | 0.012 | 0.0017 | 1.03E-12 |
| rs62176243 | A | 0.0148 | 0.0022 | 2.41E-11 |
| rs56133507 | T | -0.0131 | 0.0024 | 4.31E-08 |
| rs34234296 | A | -0.0145 | 0.002 | 2.01E-13 |
| rs262255 | T | 0.0096 | 0.0017 | 3.19E-08 |
| rs7588437 | A | -0.0165 | 0.0017 | 2.32E-22 |
| rs6716898 | A | 0.0127 | 0.0019 | 2.33E-11 |
| rs7593917 | A | -0.0115 | 0.0016 | 9.67E-13 |
| rs4482463 | A | -0.031 | 0.0031 | 4.85E-23 |
| rs11692326 | T | 0.0147 | 0.0019 | 1.69E-14 |
| rs73985439 | A | -0.0131 | 0.0021 | 1.84E-10 |
| rs7599312 | A | -0.0182 | 0.0018 | 1.52E-23 |
| rs6725931 | T | 0.0187 | 0.0024 | 2.30E-15 |
| rs4973618 | A | -0.0148 | 0.0018 | 1.25E-16 |
| rs6720868 | T | 0.0154 | 0.0018 | 1.82E-17 |
| rs9808302 | A | -0.0116 | 0.0019 | 9.05E-10 |
| rs59302296 | A | 0.0217 | 0.0032 | 9.12E-12 |
| rs6783054 | A | 0.0099 | 0.0017 | 6.22E-09 |
| rs10510419 | T | -0.0168 | 0.0023 | 2.23E-13 |
| rs2600226 | T | -0.0116 | 0.0018 | 1.42E-10 |
| rs1048637 | T | -0.0094 | 0.0017 | 2.94E-08 |
| rs4858193 | T | 0.0133 | 0.0019 | 2.44E-12 |
| rs6804842 | A | -0.0141 | 0.0016 | 7.57E-18 |
| rs11921432 | T | -0.0189 | 0.0027 | 5.38E-12 |
| rs1799923 | A | -0.0224 | 0.0026 | 1.12E-17 |
| rs28350 | A | 0.0172 | 0.0022 | 1.07E-14 |
| rs4017425 | T | -0.0118 | 0.0017 | 2.91E-12 |
| rs11919665 | A | 0.0117 | 0.002 | 7.30E-09 |
| rs11713193 | A | 0.0246 | 0.0017 | 3.02E-48 |
| rs2365389 | T | -0.0168 | 0.0016 | 6.49E-25 |
| rs6445258 | T | 0.0131 | 0.0023 | 2.48E-08 |
| rs925018 | C | -0.013 | 0.0017 | 4.33E-14 |
| rs62253188 | A | 0.0113 | 0.002 | 3.21E-08 |
| rs11915371 | A | -0.0154 | 0.0021 | 2.29E-13 |
| rs12636480 | T | 0.0128 | 0.0018 | 6.96E-13 |
| rs9827823 | T | 0.0182 | 0.0023 | 3.39E-15 |
| rs9818122 | T | -0.0228 | 0.002 | 3.97E-30 |
| rs34184235 | T | -0.0115 | 0.0019 | 2.14E-09 |
| rs11128021 | A | -0.0181 | 0.0024 | 1.63E-14 |
| rs1492014 | T | -0.0171 | 0.0017 | 1.44E-23 |
| rs1436344 | C | 0.0147 | 0.0017 | 1.07E-17 |
| rs7640424 | T | -0.0135 | 0.0018 | 1.23E-14 |
| rs17681451 | A | -0.0225 | 0.0031 | 7.37E-13 |
| rs1011613 | A | 0.0118 | 0.002 | 1.57E-09 |
| rs7631156 | A | 0.0215 | 0.0018 | 3.33E-32 |
| rs687339 | T | 0.0188 | 0.0019 | 4.32E-22 |
| rs16851483 | T | 0.0352 | 0.0034 | 4.87E-25 |
| rs355777 | C | 0.0151 | 0.0017 | 2.13E-18 |
| rs9826775 | A | 0.0155 | 0.0024 | 6.61E-11 |
| rs2047648 | A | -0.0133 | 0.002 | 7.95E-12 |
| rs12635553 | A | 0.0099 | 0.0017 | 4.10E-09 |
| rs39654 | A | -0.0163 | 0.0017 | 1.75E-21 |
| rs6443750 | T | -0.0152 | 0.0021 | 7.25E-13 |
| rs865809 | A | 0.0124 | 0.002 | 6.50E-10 |
| rs9816226 | A | -0.0315 | 0.0021 | 1.45E-50 |
| rs7616009 | A | -0.0157 | 0.0024 | 4.33E-11 |
| rs6803161 | T | 0.0107 | 0.0019 | 2.71E-08 |
| rs2051559 | T | -0.0167 | 0.0025 | 3.78E-11 |
| rs12642970 | C | -0.0109 | 0.0019 | 1.71E-08 |
| rs994596 | T | 0.013 | 0.0018 | 1.06E-12 |
| rs34811474 | A | -0.0293 | 0.0023 | 8.50E-38 |
| rs73213484 | A | 0.0208 | 0.0027 | 2.04E-14 |
| rs4261944 | T | -0.0119 | 0.0017 | 1.14E-11 |
| rs13132853 | A | 0.0148 | 0.0018 | 1.05E-16 |
| rs10938397 | A | -0.0322 | 0.0016 | 2.42E-86 |
| rs2271046 | A | -0.0115 | 0.0018 | 3.03E-10 |
| rs1492767 | T | 0.0095 | 0.0016 | 3.55E-09 |
| rs2192158 | A | 0.0137 | 0.0017 | 7.42E-16 |
| rs1346841 | A | -0.0126 | 0.0017 | 3.18E-13 |
| rs10002111 | A | 0.0125 | 0.0021 | 1.26E-09 |
| rs10033843 | A | 0.014 | 0.0021 | 1.07E-11 |
| rs72649373 | T | -0.0167 | 0.0028 | 3.20E-09 |
| rs4148155 | A | 0.0192 | 0.0026 | 1.34E-13 |
| rs4286488 | A | 0.0121 | 0.002 | 1.70E-09 |
| rs7685048 | T | -0.0101 | 0.0017 | 2.34E-09 |
| rs2241743 | A | -0.0106 | 0.0016 | 8.57E-11 |
| rs13107325 | T | 0.0468 | 0.0032 | 3.81E-47 |
| rs326893 | T | 0.0121 | 0.0017 | 1.89E-12 |
| rs7696649 | A | 0.0116 | 0.0019 | 4.73E-10 |
| rs4864201 | T | 0.0137 | 0.0017 | 4.30E-16 |
| rs1296328 | A | 0.0166 | 0.0017 | 3.49E-22 |
| rs17367750 | T | -0.0122 | 0.0018 | 1.97E-11 |
| rs3914628 | T | 0.0165 | 0.0023 | 6.91E-13 |
| rs750090 | T | 0.0113 | 0.0018 | 2.50E-10 |
| rs13110266 | A | -0.0124 | 0.0016 | 3.96E-14 |
| rs7685628 | A | 0.0101 | 0.0017 | 6.15E-09 |
| rs1522569 | T | 0.0141 | 0.0022 | 1.60E-10 |
| rs1437842 | A | -0.0106 | 0.0017 | 8.49E-10 |
| rs6850421 | A | 0.0113 | 0.0019 | 3.66E-09 |
| rs35408866 | A | 0.0159 | 0.0028 | 1.50E-08 |
| rs698147 | A | 0.0116 | 0.0017 | 9.67E-12 |
| rs6890310 | A | -0.0119 | 0.0019 | 3.29E-10 |
| rs7730004 | T | 0.0139 | 0.0018 | 1.46E-14 |
| rs116374395 | A | 0.0321 | 0.0052 | 7.05E-10 |
| rs150215901 | A | -0.0275 | 0.0049 | 1.54E-08 |
| rs4865796 | A | -0.0096 | 0.0018 | 4.21E-08 |
| rs6881799 | A | -0.0102 | 0.0017 | 3.96E-09 |
| rs4700608 | T | -0.0155 | 0.0017 | 4.32E-20 |
| rs10050620 | T | -0.013 | 0.002 | 1.20E-10 |
| rs1159692 | A | 0.0135 | 0.0017 | 5.54E-15 |
| rs2112347 | T | 0.0276 | 0.0017 | 1.17E-61 |
| rs13182474 | C | -0.0149 | 0.0018 | 1.88E-16 |
| rs2962334 | T | 0.0396 | 0.0059 | 1.78E-11 |
| rs1501673 | A | 0.0289 | 0.0025 | 2.73E-31 |
| rs2009416 | T | -0.0119 | 0.0018 | 1.44E-11 |
| rs7713317 | A | -0.0166 | 0.0018 | 1.96E-20 |
| rs6882366 | T | -0.0131 | 0.0017 | 4.39E-14 |
| rs11739877 | T | 0.0116 | 0.0018 | 4.01E-11 |
| rs6888194 | T | -0.0127 | 0.0023 | 3.58E-08 |
| rs40067 | A | -0.0252 | 0.0023 | 9.73E-29 |
| rs10478110 | A | -0.0106 | 0.0017 | 6.00E-10 |
| rs6595205 | C | 0.0112 | 0.0016 | 5.26E-12 |
| rs4572998 | T | 0.0098 | 0.0017 | 1.14E-08 |
| rs4836133 | A | 0.0132 | 0.0018 | 5.62E-14 |
| rs6886072 | T | -0.01 | 0.0017 | 4.13E-09 |
| rs13174863 | A | -0.0197 | 0.0023 | 1.94E-17 |
| rs17405603 | A | -0.0125 | 0.0019 | 4.43E-11 |
| rs2910026 | T | -0.0132 | 0.0021 | 5.69E-10 |
| rs7715256 | T | -0.0158 | 0.0016 | 3.98E-22 |
| rs7734385 | A | -0.0101 | 0.0016 | 6.08E-10 |
| rs4921301 | T | -0.0129 | 0.0024 | 4.03E-08 |
| rs2861089 | A | 0.0105 | 0.0017 | 1.55E-09 |
| rs7727781 | T | 0.0093 | 0.0017 | 4.27E-08 |
| rs2053682 | A | 0.017 | 0.0018 | 2.59E-20 |
| rs6556301 | T | -0.0113 | 0.0017 | 8.14E-11 |
| rs2228213 | A | -0.0144 | 0.0017 | 5.50E-17 |
| rs11757278 | T | 0.0133 | 0.0019 | 6.92E-13 |
| rs3806114 | A | -0.012 | 0.0018 | 1.46E-11 |
| rs767943 | A | 0.0109 | 0.0019 | 1.13E-08 |
| rs2066295 | A | 0.0142 | 0.002 | 2.29E-12 |
| rs3115667 | T | -0.0179 | 0.0019 | 5.92E-21 |
| rs2281819 | A | -0.0154 | 0.002 | 2.09E-14 |
| rs2744974 | T | 0.0261 | 0.0017 | 1.28E-51 |
| rs2436728 | A | 0.0189 | 0.0017 | 1.97E-29 |
| rs1358980 | T | -0.0129 | 0.0017 | 5.15E-15 |
| rs1554790 | C | -0.013 | 0.0017 | 2.00E-14 |
| rs2206277 | T | 0.0408 | 0.0021 | 1.82E-83 |
| rs1327259 | A | 0.0157 | 0.0017 | 1.47E-19 |
| rs6915002 | T | 0.0099 | 0.0017 | 8.71E-09 |
| rs9370410 | A | 0.0105 | 0.0019 | 2.58E-08 |
| rs2622274 | T | -0.0107 | 0.0017 | 3.23E-10 |
| rs1293037 | T | 0.0129 | 0.0022 | 5.73E-09 |
| rs6921533 | T | 0.0104 | 0.0018 | 1.37E-08 |
| rs9294260 | A | 0.014 | 0.0016 | 8.16E-18 |
| rs6909685 | T | -0.0149 | 0.0018 | 2.78E-16 |
| rs9320823 | T | -0.0165 | 0.0017 | 2.07E-21 |
| rs57989773 | T | -0.0142 | 0.0023 | 5.63E-10 |
| rs156201 | C | 0.0124 | 0.0019 | 1.65E-10 |
| rs768023 | A | 0.0161 | 0.0016 | 1.13E-22 |
| rs2357760 | A | 0.0143 | 0.0017 | 2.11E-16 |
| rs2875762 | C | 0.0129 | 0.002 | 1.07E-10 |
| rs1268065 | A | -0.01 | 0.0016 | 7.12E-10 |
| rs9375702 | T | -0.0106 | 0.0018 | 5.52E-09 |
| rs2246012 | T | -0.0161 | 0.0022 | 1.15E-13 |
| rs6922607 | A | -0.013 | 0.0022 | 1.85E-09 |
| rs765875 | T | -0.0132 | 0.0017 | 1.08E-14 |
| rs7760482 | A | -0.0102 | 0.0018 | 6.25E-09 |
| rs12527426 | A | 0.015 | 0.0019 | 5.54E-16 |
| rs9478496 | T | -0.0157 | 0.0023 | 8.17E-12 |
| rs13191362 | A | 0.0235 | 0.0025 | 4.08E-21 |
| rs9458814 | T | -0.0112 | 0.002 | 2.33E-08 |
| rs6950388 | A | 0.0135 | 0.0022 | 1.59E-09 |
| rs4721089 | T | 0.0167 | 0.0023 | 5.60E-13 |
| rs6463489 | T | 0.0167 | 0.0026 | 2.50E-10 |
| rs4307239 | A | -0.0115 | 0.0017 | 1.47E-11 |
| rs213518 | T | -0.0153 | 0.0024 | 2.49E-10 |
| rs215669 | A | -0.0149 | 0.0017 | 8.94E-18 |
| rs2108719 | A | 0.0107 | 0.0019 | 2.28E-08 |
| rs217433 | T | -0.0115 | 0.0021 | 3.00E-08 |
| rs2289379 | T | -0.0137 | 0.0018 | 7.02E-15 |
| rs10499694 | A | 0.013 | 0.0016 | 1.29E-15 |
| rs254927 | T | 0.0107 | 0.0019 | 2.19E-08 |
| rs11772246 | T | 0.0145 | 0.0022 | 3.69E-11 |
| rs17207196 | T | -0.022 | 0.0017 | 1.58E-36 |
| rs17149254 | T | 0.0238 | 0.0023 | 2.99E-25 |
| rs1965529 | A | 0.0161 | 0.0022 | 7.66E-14 |
| rs274628 | A | -0.0102 | 0.0018 | 1.36E-08 |
| rs2283006 | A | 0.0132 | 0.0017 | 8.06E-15 |
| rs13240600 | A | 0.018 | 0.0022 | 7.90E-16 |
| rs1721447 | T | -0.01 | 0.0017 | 3.79E-09 |
| rs2396625 | A | -0.0176 | 0.0017 | 2.81E-24 |
| rs13245051 | A | 0.015 | 0.0017 | 1.12E-18 |
| rs10261050 | T | 0.0113 | 0.0017 | 4.53E-11 |
| rs2283093 | T | 0.0121 | 0.0021 | 1.10E-08 |
| rs7802342 | T | -0.0124 | 0.0019 | 6.23E-11 |
| rs11525873 | T | 0.0232 | 0.0032 | 2.98E-13 |
| rs11773362 | T | -0.0105 | 0.0018 | 6.39E-09 |
| rs2907948 | A | -0.0145 | 0.0019 | 1.95E-14 |
| rs56211164 | A | -0.0128 | 0.0022 | 8.90E-09 |
| rs1700082 | C | 0.0094 | 0.0017 | 3.38E-08 |
| rs4240673 | T | 0.0175 | 0.0016 | 1.58E-26 |
| rs13263601 | A | -0.0145 | 0.0018 | 4.87E-16 |
| rs10110189 | T | -0.0156 | 0.0028 | 2.20E-08 |
| rs10101364 | T | 0.012 | 0.0018 | 5.61E-11 |
| rs73225274 | A | -0.0156 | 0.0028 | 2.33E-08 |
| rs13249650 | A | 0.011 | 0.0019 | 1.02E-08 |
| rs1421334 | A | 0.0135 | 0.0017 | 3.11E-15 |
| rs2466103 | T | -0.0121 | 0.0018 | 8.00E-12 |
| rs6468266 | A | -0.0113 | 0.0017 | 7.71E-11 |
| rs12681792 | A | 0.015 | 0.0021 | 2.88E-12 |
| rs12334877 | A | -0.0144 | 0.0021 | 2.17E-11 |
| rs1808629 | A | -0.0202 | 0.002 | 2.19E-23 |
| rs17405819 | T | 0.0211 | 0.0018 | 6.04E-33 |
| rs2196618 | A | -0.0137 | 0.0019 | 1.49E-12 |
| rs1504804 | A | -0.0107 | 0.0018 | 4.74E-09 |
| rs56862898 | A | 0.0132 | 0.0024 | 2.35E-08 |
| rs12680842 | A | 0.0142 | 0.0017 | 3.41E-16 |
| rs1383592 | A | 0.0122 | 0.0021 | 4.92E-09 |
| rs3808477 | T | -0.0182 | 0.0019 | 8.73E-22 |
| rs72673947 | A | -0.0223 | 0.0031 | 4.11E-13 |
| rs6470144 | T | 0.0098 | 0.0018 | 3.06E-08 |
| rs7842934 | T | -0.0178 | 0.0031 | 1.55E-08 |
| rs16906838 | T | -0.0247 | 0.004 | 4.31E-10 |
| rs11782074 | T | 0.0124 | 0.0018 | 4.24E-12 |
| rs10099330 | A | -0.0119 | 0.0017 | 3.22E-12 |
| rs10757259 | A | -0.0113 | 0.002 | 2.74E-08 |
| rs10975933 | C | 0.0114 | 0.0018 | 2.07E-10 |
| rs1948080 | T | 0.0136 | 0.0018 | 1.13E-14 |
| rs10961649 | T | 0.0104 | 0.0018 | 1.51E-08 |
| rs4740619 | T | 0.0189 | 0.0016 | 3.15E-31 |
| rs10962550 | C | 0.0186 | 0.0022 | 5.68E-17 |
| rs10811868 | A | -0.01 | 0.0018 | 3.18E-08 |
| rs10968114 | A | 0.0113 | 0.0017 | 3.30E-11 |
| rs1412235 | C | 0.0237 | 0.0017 | 2.28E-42 |
| rs7030297 | A | -0.0102 | 0.0017 | 2.07E-09 |
| rs6476617 | A | -0.0149 | 0.0017 | 1.53E-17 |
| rs2134858 | T | -0.0117 | 0.0017 | 5.87E-12 |
| rs7861160 | T | 0.0095 | 0.0017 | 3.68E-08 |
| rs1999433 | T | -0.0107 | 0.0017 | 2.90E-10 |
| rs2777768 | A | 0.0119 | 0.0019 | 6.38E-10 |
| rs7357754 | A | -0.012 | 0.0017 | 1.81E-12 |
| rs10992867 | A | 0.0162 | 0.0019 | 3.20E-17 |
| rs7025938 | C | -0.0162 | 0.0018 | 1.47E-19 |
| rs7024334 | T | 0.0135 | 0.002 | 4.71E-12 |
| rs1928295 | T | 0.0134 | 0.0016 | 2.23E-16 |
| rs1877875 | T | -0.0109 | 0.0017 | 2.51E-10 |
| rs13298487 | T | 0.0115 | 0.002 | 1.43E-08 |
| rs10733682 | A | 0.0148 | 0.0016 | 1.76E-19 |
| rs2267958 | A | -0.013 | 0.0018 | 2.01E-13 |
| rs4740383 | A | 0.0131 | 0.0018 | 7.03E-14 |
| rs10858334 | C | -0.0148 | 0.0025 | 4.91E-09 |
| rs7907470 | A | -0.0177 | 0.0031 | 7.36E-09 |
| rs7893571 | T | 0.0125 | 0.0018 | 5.83E-12 |
| rs76638898 | A | -0.0376 | 0.0065 | 8.15E-09 |
| rs7084454 | A | 0.0198 | 0.0018 | 4.51E-27 |
| rs10829164 | T | 0.0151 | 0.0024 | 2.37E-10 |
| rs4097319 | T | 0.0107 | 0.0017 | 4.32E-10 |
| rs12765914 | T | 0.0226 | 0.0031 | 1.96E-13 |
| rs1624134 | C | 0.01 | 0.0017 | 6.51E-09 |
| rs12259464 | A | 0.0109 | 0.0017 | 1.48E-10 |
| rs7070670 | T | -0.0126 | 0.0021 | 7.14E-10 |
| rs10761785 | T | -0.0133 | 0.0016 | 3.47E-16 |
| rs12098284 | T | 0.0184 | 0.0026 | 9.87E-13 |
| rs3213967 | T | 0.0122 | 0.0022 | 3.60E-08 |
| rs12243061 | T | 0.0105 | 0.0018 | 5.82E-09 |
| rs7899106 | A | -0.0327 | 0.0037 | 1.72E-18 |
| rs10788494 | C | 0.0132 | 0.0017 | 5.51E-15 |
| rs147568678 | T | 0.0134 | 0.0023 | 3.42E-09 |
| rs2439823 | A | -0.0165 | 0.0017 | 6.51E-22 |
| rs17094222 | T | -0.0173 | 0.002 | 4.04E-18 |
| rs11594179 | T | -0.0109 | 0.0019 | 2.16E-08 |
| rs79780963 | T | 0.0244 | 0.0035 | 2.63E-12 |
| rs7903146 | T | -0.0178 | 0.0018 | 1.67E-23 |
| rs2257791 | A | -0.0139 | 0.002 | 1.63E-12 |
| rs845084 | A | 0.0136 | 0.0019 | 3.22E-12 |
| rs17636031 | T | -0.0154 | 0.0018 | 3.87E-17 |
| rs4880341 | T | -0.013 | 0.0017 | 3.06E-14 |
| rs11246136 | A | -0.0167 | 0.003 | 2.03E-08 |
| rs12421848 | A | -0.0141 | 0.0019 | 3.99E-13 |
| rs4256980 | C | -0.0187 | 0.0017 | 8.63E-29 |
| rs900144 | T | 0.0148 | 0.0017 | 1.53E-18 |
| rs2074314 | T | 0.0105 | 0.0017 | 1.37E-09 |
| rs6265 | T | -0.0413 | 0.0021 | 7.40E-89 |
| rs570463 | A | -0.0121 | 0.0018 | 4.33E-11 |
| rs11030618 | T | 0.011 | 0.0017 | 1.67E-10 |
| rs2065418 | T | 0.0139 | 0.0018 | 6.07E-15 |
| rs2862996 | T | -0.0216 | 0.0017 | 3.60E-35 |
| rs10742752 | T | -0.0123 | 0.0017 | 1.20E-13 |
| rs118081010 | T | 0.0518 | 0.0075 | 5.28E-12 |
| rs7124681 | A | 0.0257 | 0.0016 | 3.96E-55 |
| rs6591407 | A | -0.0124 | 0.0021 | 3.58E-09 |
| rs2244625 | A | 0.0103 | 0.0017 | 2.18E-09 |
| rs7102454 | T | -0.0168 | 0.0018 | 3.84E-21 |
| rs592483 | T | -0.0137 | 0.0017 | 1.53E-16 |
| rs12282785 | A | -0.0157 | 0.0023 | 1.43E-11 |
| rs10899469 | T | 0.0122 | 0.0022 | 3.56E-08 |
| rs349088 | A | -0.013 | 0.0017 | 3.50E-14 |
| rs647248 | A | 0.0108 | 0.0017 | 3.96E-10 |
| rs3019466 | T | -0.0128 | 0.0024 | 4.91E-08 |
| rs2605603 | A | -0.0103 | 0.0016 | 2.04E-10 |
| rs2513999 | A | -0.0154 | 0.0026 | 3.07E-09 |
| rs12286929 | A | -0.0177 | 0.0016 | 1.93E-27 |
| rs76942203 | A | 0.0263 | 0.0041 | 9.08E-11 |
| rs3825061 | T | 0.014 | 0.0017 | 6.15E-16 |
| rs11218510 | A | -0.014 | 0.002 | 6.79E-13 |
| rs35483388 | T | 0.0125 | 0.002 | 2.61E-10 |
| rs7944782 | T | -0.0144 | 0.0017 | 3.61E-17 |
| rs2007518 | A | -0.013 | 0.0017 | 1.96E-14 |
| rs1941213 | A | 0.0108 | 0.0019 | 1.59E-08 |
| rs329651 | T | 0.016 | 0.0021 | 2.13E-14 |
| rs12364470 | T | -0.0187 | 0.0022 | 2.18E-17 |
| rs11611246 | T | 0.0223 | 0.002 | 2.04E-28 |
| rs765125 | T | -0.0098 | 0.0017 | 1.27E-08 |
| rs10772983 | T | -0.01 | 0.0016 | 6.21E-10 |
| rs11044430 | A | 0.0161 | 0.0023 | 3.77E-12 |
| rs11046972 | T | 0.0176 | 0.0032 | 3.72E-08 |
| rs10842240 | C | 0.0195 | 0.0025 | 1.21E-14 |
| rs11170468 | A | 0.013 | 0.0019 | 1.12E-11 |
| rs1350430 | T | -0.0125 | 0.0017 | 2.08E-13 |
| rs1126930 | C | 0.0341 | 0.0053 | 1.00E-10 |
| rs7138803 | A | 0.0297 | 0.0017 | 3.10E-71 |
| rs2271189 | A | -0.0141 | 0.0018 | 9.26E-16 |
| rs4267103 | T | -0.015 | 0.0024 | 5.54E-10 |
| rs650198 | T | -0.0137 | 0.0019 | 6.43E-13 |
| rs11115176 | T | 0.0131 | 0.0019 | 6.79E-12 |
| rs10506971 | A | -0.0142 | 0.0017 | 4.69E-17 |
| rs55966114 | T | 0.0146 | 0.0024 | 2.47E-09 |
| rs2712666 | A | -0.0144 | 0.002 | 1.54E-13 |
| rs6539064 | C | 0.0194 | 0.0019 | 1.14E-23 |
| rs10161401 | A | 0.0108 | 0.002 | 3.63E-08 |
| rs1860561 | A | 0.0159 | 0.0019 | 1.71E-16 |
| rs11066188 | A | -0.0114 | 0.0016 | 3.05E-12 |
| rs2707183 | T | -0.0093 | 0.0017 | 4.89E-08 |
| rs11615578 | T | 0.0117 | 0.002 | 3.04E-09 |
| rs12369179 | T | -0.034 | 0.0031 | 2.32E-28 |
| rs11060853 | A | -0.0107 | 0.0019 | 3.10E-08 |
| rs11614340 | T | -0.0117 | 0.0018 | 1.87E-10 |
| rs9512648 | A | 0.0095 | 0.0017 | 4.11E-08 |
| rs7323 | C | -0.0166 | 0.0019 | 2.18E-18 |
| rs1933437 | A | -0.0143 | 0.0017 | 2.36E-16 |
| rs9595908 | T | 0.0154 | 0.0017 | 3.73E-20 |
| rs9603697 | T | 0.0134 | 0.0018 | 1.69E-13 |
| rs12429545 | A | 0.0313 | 0.0024 | 1.42E-37 |
| rs962796 | T | 0.014 | 0.0021 | 1.88E-11 |
| rs9569777 | T | -0.0201 | 0.0021 | 3.43E-21 |
| rs9527895 | T | -0.0158 | 0.0023 | 3.69E-12 |
| rs8181823 | A | -0.0125 | 0.002 | 4.36E-10 |
| rs9599161 | T | 0.0098 | 0.0016 | 2.32E-09 |
| rs1441264 | A | 0.0174 | 0.0017 | 7.60E-25 |
| rs9531786 | C | -0.0111 | 0.0018 | 4.11E-10 |
| rs77432547 | A | -0.017 | 0.0021 | 1.44E-15 |
| rs1927790 | T | -0.014 | 0.0016 | 1.57E-17 |
| rs7334078 | T | 0.0139 | 0.0019 | 1.45E-13 |
| rs9559022 | A | -0.0121 | 0.0022 | 4.86E-08 |
| rs9522183 | T | -0.0135 | 0.0019 | 2.10E-12 |
| rs12868881 | A | 0.0138 | 0.0017 | 1.40E-15 |
| rs10132280 | A | -0.0214 | 0.0018 | 2.28E-33 |
| rs4981693 | A | 0.0202 | 0.002 | 7.89E-24 |
| rs225882 | T | 0.0113 | 0.0018 | 8.56E-10 |
| rs1958898 | C | -0.0146 | 0.0021 | 4.23E-12 |
| rs4900714 | T | 0.015 | 0.0017 | 1.00E-18 |
| rs217669 | T | -0.0172 | 0.0021 | 6.27E-16 |
| rs4430672 | T | 0.0124 | 0.0021 | 5.00E-09 |
| rs3902951 | T | -0.0134 | 0.0019 | 2.88E-12 |
| rs17182027 | A | -0.011 | 0.0017 | 1.46E-10 |
| rs7144011 | T | 0.0263 | 0.002 | 2.37E-40 |
| rs12888545 | A | -0.0133 | 0.002 | 1.76E-11 |
| rs1951455 | T | -0.0148 | 0.0019 | 6.05E-15 |
| rs942066 | A | -0.0202 | 0.002 | 2.36E-24 |
| rs12147845 | T | 0.0187 | 0.0027 | 4.97E-12 |
| rs7161194 | A | 0.019 | 0.0019 | 2.23E-24 |
| rs4906263 | C | -0.0176 | 0.0018 | 8.11E-23 |
| rs2273175 | T | -0.0121 | 0.0018 | 3.69E-11 |
| rs12594043 | C | 0.0102 | 0.0017 | 1.31E-09 |
| rs7172627 | A | -0.0114 | 0.0017 | 1.81E-11 |
| rs11636611 | T | 0.0104 | 0.0017 | 8.85E-10 |
| rs9944219 | A | -0.0119 | 0.0017 | 8.14E-13 |
| rs12912198 | T | -0.01 | 0.0018 | 3.44E-08 |
| rs6493498 | T | 0.0137 | 0.0016 | 4.84E-17 |
| rs10518694 | A | 0.0144 | 0.0024 | 3.43E-09 |
| rs12439632 | C | 0.0145 | 0.0023 | 3.68E-10 |
| rs339991 | A | -0.0125 | 0.0017 | 3.51E-13 |
| rs1559673 | A | 0.0359 | 0.0049 | 2.21E-13 |
| rs2241423 | A | -0.0298 | 0.0019 | 3.60E-54 |
| rs7171864 | A | 0.0132 | 0.0017 | 2.60E-14 |
| rs2470893 | T | 0.0107 | 0.0017 | 9.43E-10 |
| rs56151256 | A | 0.0166 | 0.0022 | 6.19E-14 |
| rs12914623 | C | -0.0159 | 0.0019 | 2.00E-16 |
| rs11633626 | A | -0.0157 | 0.0018 | 7.27E-19 |
| rs12439829 | A | -0.0105 | 0.0018 | 2.28E-09 |
| rs2715423 | A | -0.0115 | 0.0019 | 1.90E-09 |
| rs214249 | T | 0.0138 | 0.0017 | 2.72E-15 |
| rs12448257 | A | 0.0161 | 0.002 | 9.04E-16 |
| rs879620 | T | 0.0226 | 0.0017 | 8.45E-39 |
| rs2058527 | T | -0.0115 | 0.0019 | 1.80E-09 |
| rs9926784 | T | 0.0237 | 0.0021 | 1.08E-30 |
| rs194809 | A | 0.0126 | 0.0022 | 4.86E-09 |
| rs2342892 | T | 0.0126 | 0.0017 | 1.33E-13 |
| rs7498665 | A | -0.0285 | 0.0017 | 1.14E-66 |
| rs3814883 | T | 0.0227 | 0.0017 | 1.47E-40 |
| rs2193101 | C | 0.0142 | 0.0021 | 3.33E-11 |
| rs16952482 | T | -0.0151 | 0.0027 | 3.06E-08 |
| rs9937053 | A | 0.0721 | 0.0016 | <1E-300 |
| rs8057911 | T | 0.0125 | 0.0023 | 3.66E-08 |
| rs889398 | T | -0.0195 | 0.0016 | 3.23E-32 |
| rs35949039 | T | -0.0215 | 0.0031 | 6.30E-12 |
| rs12926250 | T | 0.018 | 0.0028 | 1.78E-10 |
| rs756717 | A | -0.0134 | 0.0017 | 2.38E-15 |
| rs11644382 | A | 0.0102 | 0.0018 | 3.04E-08 |
| rs12922346 | C | 0.0133 | 0.002 | 1.48E-11 |
| rs7206608 | C | -0.013 | 0.0018 | 1.20E-12 |
| rs3923783 | A | -0.0222 | 0.0022 | 4.12E-23 |
| rs11078883 | C | -0.0126 | 0.0018 | 1.30E-12 |
| rs7774 | A | 0.0128 | 0.0021 | 4.22E-10 |
| rs1075901 | T | -0.0118 | 0.0016 | 4.43E-13 |
| rs4986044 | T | -0.0177 | 0.0016 | 1.27E-27 |
| rs11651715 | A | 0.0111 | 0.0019 | 8.22E-09 |
| rs12150665 | T | 0.0168 | 0.0016 | 1.74E-24 |
| rs6607337 | T | -0.0124 | 0.0019 | 2.55E-11 |
| rs56161855 | A | -0.0233 | 0.0028 | 1.41E-16 |
| rs9299 | T | 0.0119 | 0.0018 | 1.87E-11 |
| rs11655587 | T | -0.021 | 0.002 | 6.87E-26 |
| rs11649864 | A | 0.0192 | 0.003 | 2.52E-10 |
| rs12602912 | T | 0.0166 | 0.002 | 2.90E-16 |
| rs312750 | A | 0.0097 | 0.0016 | 2.57E-09 |
| rs12939549 | A | 0.018 | 0.0016 | 3.68E-28 |
| rs35867081 | A | 0.015 | 0.0019 | 6.65E-15 |
| rs8097672 | A | -0.0209 | 0.0024 | 3.26E-18 |
| rs478707 | T | -0.015 | 0.0024 | 1.98E-10 |
| rs891387 | T | 0.0208 | 0.0017 | 9.26E-35 |
| rs1945160 | A | -0.0104 | 0.0018 | 5.65E-09 |
| rs1941696 | A | 0.0112 | 0.0017 | 4.74E-11 |
| rs474605 | A | -0.0125 | 0.0017 | 2.72E-13 |
| rs1356506 | T | 0.0137 | 0.0018 | 8.39E-15 |
| rs2612576 | A | -0.0108 | 0.0019 | 8.49E-09 |
| rs4534948 | A | -0.0093 | 0.0017 | 4.87E-08 |
| rs9951619 | T | -0.0152 | 0.0019 | 2.33E-15 |
| rs6567160 | T | -0.0552 | 0.0019 | 7.82E-184 |
| rs17066856 | T | 0.035 | 0.0027 | 7.10E-38 |
| rs66595146 | A | 0.0135 | 0.002 | 9.09E-12 |
| rs17783165 | T | -0.0128 | 0.0017 | 2.74E-13 |
| rs11150911 | A | 0.0118 | 0.0018 | 3.72E-11 |
| rs12981256 | A | 0.0151 | 0.0017 | 1.43E-18 |
| rs45486197 | A | 0.0279 | 0.004 | 1.80E-12 |
| rs895330 | C | 0.0196 | 0.0022 | 1.59E-19 |
| rs273512 | T | 0.0156 | 0.0018 | 4.48E-19 |
| rs17724992 | A | 0.0172 | 0.0018 | 5.23E-21 |
| rs7258722 | A | -0.02 | 0.0017 | 1.81E-30 |
| rs12611148 | A | -0.0137 | 0.0024 | 1.42E-08 |
| rs12462975 | A | 0.0193 | 0.0018 | 1.47E-25 |
| rs7245985 | T | 0.0119 | 0.0021 | 1.88E-08 |
| rs11882409 | A | 0.0121 | 0.0019 | 3.31E-10 |
| rs185350 | T | 0.0137 | 0.0016 | 9.10E-17 |
| rs429358 | T | 0.0257 | 0.0026 | 2.60E-22 |
| rs11672660 | T | -0.0338 | 0.0021 | 6.83E-60 |
| rs9304665 | A | 0.023 | 0.002 | 2.20E-31 |
| rs3764625 | T | 0.0096 | 0.0017 | 2.42E-08 |
| rs1884389 | T | -0.0108 | 0.0017 | 3.72E-10 |
| rs4813619 | T | -0.0106 | 0.0018 | 1.70E-09 |
| rs1884897 | A | -0.0184 | 0.0017 | 2.70E-28 |
| rs2423668 | T | 0.0106 | 0.0018 | 7.84E-09 |
| rs852056 | T | 0.0123 | 0.0019 | 2.15E-10 |
| rs1409818 | T | 0.0195 | 0.0028 | 2.59E-12 |
| rs8122855 | A | 0.0137 | 0.0018 | 4.11E-14 |
| rs4812405 | A | -0.0202 | 0.0033 | 1.28E-09 |
| rs742748 | T | -0.0113 | 0.0017 | 1.13E-11 |
| rs79186842 | A | 0.0202 | 0.0028 | 2.60E-13 |
| rs17806224 | A | -0.026 | 0.0022 | 7.91E-32 |
| rs1304549 | A | -0.0118 | 0.0021 | 1.35E-08 |
| rs6010784 | T | 0.0106 | 0.0016 | 6.91E-11 |
| rs6512302 | C | 0.0134 | 0.002 | 1.52E-11 |
| rs2064044 | A | -0.0123 | 0.0021 | 1.02E-08 |
| rs2832283 | A | 0.0115 | 0.002 | 4.72E-09 |
| rs13047416 | C | 0.0152 | 0.0018 | 6.93E-18 |
| rs8134638 | T | -0.0133 | 0.002 | 1.57E-11 |
| rs8126575 | T | 0.0152 | 0.0025 | 5.88E-10 |
| rs427943 | A | -0.0177 | 0.0017 | 3.60E-25 |
| rs140733155 | A | -0.0559 | 0.0094 | 2.97E-09 |
| rs11538 | A | -0.0138 | 0.0023 | 1.09E-09 |
| rs2238799 | A | 0.0102 | 0.0018 | 8.65E-09 |
| rs12628891 | T | -0.0115 | 0.0019 | 5.85E-10 |
| rs12628051 | T | 0.0161 | 0.0018 | 2.90E-19 |
| rs28489620 | A | -0.0151 | 0.0021 | 1.42E-12 |
| rs9615337 | C | -0.011 | 0.002 | 2.05E-08 |

# **Supplementary Table 5. Genetic variants that were used as proxies for liability to chronic obstructive pulmonary disease (after clumping).**

| Variant | Effect Allele | Effect | Standard Error | P |
| --- | --- | --- | --- | --- |
| rs72673419 | T | 0.131028 | 0.022392 | 4.00E-09 |
| rs629619 | T | 0.076961 | 0.011758 | 2.90E-10 |
| rs10929386 | C | 0.058269 | 0.009628 | 9.10E-09 |
| rs62259026 | C | 0.067659 | 0.011979 | 2.40E-08 |
| rs4585380 | G | 0.067659 | 0.009538 | 3.40E-09 |
| rs12519165 | A | 0.067659 | 0.009538 | 1.10E-09 |
| rs646695 | C | 0.076961 | 0.011867 | 4.60E-11 |
| rs2040732 | C | 0.058269 | 0.009628 | 6.90E-09 |
| rs1570221 | A | 0.058269 | 0.009628 | 2.20E-08 |
| rs4757118 | T | 0.058269 | 0.009628 | 3.80E-09 |
| rs9525927 | G | 0.076961 | 0.011867 | 2.80E-09 |
| rs72731149 | G | 0.113329 | 0.018229 | 8.30E-09 |
| rs10152300 | G | 0.076961 | 0.011758 | 4.20E-12 |
| rs955277 | T | 0.067659 | 0.009538 | 1.90E-10 |
| rs12466981 | C | 0.058269 | 0.011979 | 4.90E-08 |
| rs2442776 | G | 0.086178 | 0.014046 | 2.00E-10 |
| rs4093840 | A | 0.058269 | 0.009628 | 3.90E-10 |
| rs62375246 | A | 0.058269 | 0.011979 | 2.20E-08 |
| rs979453 | G | 0.058269 | 0.009628 | 1.40E-08 |
| rs1334576 | A | 0.058269 | 0.009628 | 1.20E-08 |
| rs9350191 | T | 0.113329 | 0.013669 | 5.10E-14 |
| rs2284174 | C | 0.113329 | 0.01134 | 2.10E-21 |
| rs674621 | C | 0.058269 | 0.009628 | 7.60E-09 |
| rs9329170 | C | 0.09531 | 0.013918 | 3.60E-10 |
| rs10760580 | G | 0.067659 | 0.011867 | 1.20E-10 |
| rs8044657 | G | 0.10436 | 0.018394 | 1.10E-08 |
| rs62065216 | A | 0.058269 | 0.009628 | 8.10E-09 |
| rs4660861 | G | 0.058269 | 0.009628 | 4.40E-08 |
| rs7650602 | C | 0.058269 | 0.009628 | 4.90E-08 |
| rs34651 | C | 0.10436 | 0.018394 | 3.00E-08 |
| rs798565 | G | 0.067659 | 0.011979 | 3.90E-09 |
| rs7866939 | C | 0.058269 | 0.009628 | 1.70E-08 |
| rs7958945 | G | 0.058269 | 0.011979 | 1.00E-09 |
| rs72626215 | G | 0.067659 | 0.011867 | 1.70E-09 |
| rs73158393 | C | 0.067659 | 0.009538 | 7.70E-09 |
| rs9435731 | A | 0.058269 | 0.009628 | 6.60E-10 |
| rs76841360 | A | 0.076961 | 0.011867 | 5.00E-10 |
| rs3009947 | C | 0.058269 | 0.009628 | 4E-09 |
| rs11118406 | T | 0.076961 | 0.011867 | 4.10E-11 |
| rs11579382 | C | 0.058269 | 0.009628 | 6.50E-09 |
| rs72902175 | T | 0.086178 | 0.014046 | 1.6E-08 |
| rs2571445 | A | 0.067659 | 0.009538 | 3.40E-12 |
| rs16825267 | C | 0.173953 | 0.019222 | 1.80E-20 |
| rs62191105 | C | 0.086178 | 0.011651 | 2.60E-12 |
| rs1529672 | C | 0.086178 | 0.014046 | 2.50E-11 |
| rs13073544 | C | 0.058269 | 0.011979 | 2E-08 |
| rs17759204 | G | 0.067659 | 0.011867 | 8.80E-11 |
| rs2955083 | A | 0.122218 | 0.015738 | 3.50E-15 |
| rs7642001 | A | 0.076961 | 0.009449 | 1.10E-14 |
| rs7671261 | A | 0.086178 | 0.009363 | 1.40E-18 |
| rs34712979 | A | 0.165514 | 0.010765 | 3.00E-46 |
| rs13140176 | A | 0.165514 | 0.008648 | 4.10E-59 |
| rs1551943 | A | 0.076961 | 0.011867 | 5.80E-10 |
| rs153916 | T | 0.058269 | 0.009628 | 6.30E-10 |
| rs10037493 | C | 0.122218 | 0.01134 | 2.60E-33 |
| rs10866659 | G | 0.086178 | 0.009363 | 1.20E-16 |
| rs13198656 | T | 0.058269 | 0.009628 | 1.20E-09 |
| rs2070600 | C | 0.19062 | 0.023304 | 1.10E-17 |
| rs2806356 | C | 0.09531 | 0.011545 | 2.90E-15 |
| rs9399401 | T | 0.14842 | 0.011045 | 1.60E-40 |
| rs2897075 | C | 0.067659 | 0.011979 | 7.30E-10 |
| rs10114763 | T | 0.067659 | 0.009538 | 8.70E-13 |
| rs156394 | T | 0.067659 | 0.009538 | 3.80E-12 |
| rs803923 | A | 0.058269 | 0.009628 | 2.7E-08 |
| rs7068966 | C | 0.09531 | 0.009277 | 6.20E-23 |
| rs2579762 | C | 0.058269 | 0.011979 | 2.60E-10 |
| rs721917 | G | 0.058269 | 0.009628 | 2.2E-08 |
| rs117261012 | G | 0.086178 | 0.014046 | 6.90E-10 |
| rs11049386 | T | 0.058269 | 0.011979 | 2.7E-08 |
| rs7307510 | C | 0.076961 | 0.011867 | 2.60E-09 |
| rs72699855 | G | 0.076961 | 0.014176 | 4.80E-09 |
| rs1441358 | G | 0.122218 | 0.009031 | 7.40E-33 |
| rs55676755 | G | 0.10436 | 0.011441 | 2.70E-26 |
| rs56134392 | C | 0.058269 | 0.009628 | 4.5E-08 |
| rs4888379 | T | 0.09531 | 0.009277 | 5.90E-21 |
| rs8080772 | T | 0.058269 | 0.009628 | 1.4E-08 |
| rs34727469 | T | 0.086178 | 0.014046 | 1.1E-08 |
| rs12373142 | G | 0.076961 | 0.011867 | 9.90E-10 |
| rs11655567 | C | 0.058269 | 0.009628 | 1.90E-09 |
| rs647097 | C | 0.076961 | 0.011867 | 1.00E-11 |
| rs2096468 | A | 0.058269 | 0.009628 | 4.00E-08 |
| rs9617650 | G | 0.076961 | 0.011758 | 4.40E-10 |

# **Supplementary Table 6. Genetic variants that were used as proxies for lifetime smoking index (after clumping).**

| Variant | Effect Allele | Effect | Standard Error | P |
| --- | --- | --- | --- | --- |
| rs1193237 | G | -0.00776 | 0.001397 | 2.80E-08 |
| rs4949465 | T | -0.01161 | 0.002058 | 1.70E-08 |
| rs549845 | G | 0.011261 | 0.001509 | 8.30E-14 |
| rs1933270 | T | 0.009218 | 0.001438 | 1.50E-10 |
| rs7528604 | G | 0.009653 | 0.001401 | 5.70E-12 |
| rs11210229 | A | 0.011702 | 0.001423 | 2.00E-16 |
| rs7553348 | G | 0.009634 | 0.001396 | 5.20E-12 |
| rs10922907 | A | 0.010175 | 0.001395 | 3.00E-13 |
| rs1931263 | G | -0.00761 | 0.001386 | 4.00E-08 |
| rs7519626 | C | 0.00842 | 0.001479 | 1.20E-08 |
| rs9435340 | T | 0.008348 | 0.001464 | 1.20E-08 |
| rs10918701 | G | 0.008024 | 0.001432 | 2.10E-08 |
| rs2867112 | T | 0.014783 | 0.001887 | 4.80E-15 |
| rs6741228 | T | 0.007933 | 0.001404 | 1.60E-08 |
| rs62135536 | C | 0.024344 | 0.003962 | 8.00E-10 |
| rs7569203 | A | -0.01076 | 0.0015 | 7.40E-13 |
| rs13016665 | C | -0.00849 | 0.001412 | 1.80E-09 |
| rs4671357 | T | -0.00944 | 0.00139 | 1.10E-11 |
| rs359243 | T | -0.00872 | 0.001425 | 9.50E-10 |
| rs2678670 | A | 0.008731 | 0.001388 | 3.10E-10 |
| rs62155874 | A | -0.01691 | 0.002085 | 5.20E-16 |
| rs3811038 | T | -0.00954 | 0.001557 | 8.90E-10 |
| rs2890772 | G | -0.0137 | 0.001407 | 2.10E-22 |
| rs62175972 | T | 0.021746 | 0.003857 | 1.70E-08 |
| rs3769949 | T | -0.00826 | 0.001386 | 2.50E-09 |
| rs13009008 | A | 0.008633 | 0.001473 | 4.60E-09 |
| rs4473348 | A | -0.01043 | 0.001597 | 6.40E-11 |
| rs12623702 | A | -0.00977 | 0.001428 | 7.70E-12 |
| rs6779302 | G | -0.00875 | 0.001439 | 1.20E-09 |
| rs6778080 | T | 0.011114 | 0.001566 | 1.30E-12 |
| rs775758 | A | 0.008014 | 0.001403 | 1.10E-08 |
| rs421983 | T | 0.008707 | 0.001386 | 3.30E-10 |
| rs326341 | G | 0.009435 | 0.001392 | 1.20E-11 |
| rs73220544 | A | -0.01082 | 0.001913 | 1.50E-08 |
| rs9842947 | C | -0.00877 | 0.001481 | 3.10E-09 |
| rs624833 | T | 0.009286 | 0.001504 | 6.60E-10 |
| rs61796681 | A | -0.01342 | 0.002448 | 4.20E-08 |
| rs317021 | T | -0.01157 | 0.001791 | 1.10E-10 |
| rs72678864 | G | 0.012383 | 0.001839 | 1.60E-11 |
| rs17576594 | G | 0.01095 | 0.001552 | 1.70E-12 |
| rs11948770 | T | -0.01024 | 0.001645 | 4.90E-10 |
| rs71627581 | G | 0.013257 | 0.002199 | 1.60E-09 |
| rs10052591 | T | 0.0084 | 0.001402 | 2.10E-09 |
| rs2080870 | A | 0.008633 | 0.001583 | 4.90E-08 |
| rs4571506 | C | 0.007877 | 0.001392 | 1.50E-08 |
| rs4957528 | A | -0.01012 | 0.001722 | 4.20E-09 |
| rs329120 | C | 0.009657 | 0.001405 | 6.30E-12 |
| rs986391 | G | 0.011139 | 0.001438 | 9.40E-15 |
| rs13153393 | A | -0.01375 | 0.002173 | 2.50E-10 |
| rs245774 | A | -0.00902 | 0.00156 | 7.40E-09 |
| rs6935954 | A | 0.009582 | 0.001402 | 8.20E-12 |
| rs2254710 | C | 0.008996 | 0.001631 | 3.50E-08 |
| rs2894808 | T | -0.0153 | 0.002591 | 3.50E-09 |
| rs12202536 | A | -0.00823 | 0.001386 | 2.80E-09 |
| rs7766610 | C | 0.012584 | 0.001793 | 2.20E-12 |
| rs1922018 | C | 0.010033 | 0.001438 | 3.00E-12 |
| rs10226228 | A | -0.01141 | 0.001437 | 2.00E-15 |
| rs11768481 | C | 0.009012 | 0.001475 | 9.90E-10 |
| rs6962772 | A | 0.011064 | 0.001916 | 7.80E-09 |
| rs10282292 | C | 0.008962 | 0.001447 | 5.90E-10 |
| rs2401924 | G | 0.010572 | 0.001389 | 2.70E-14 |
| rs7807019 | A | -0.01042 | 0.001391 | 6.70E-14 |
| rs6957896 | C | -0.00758 | 0.001387 | 4.50E-08 |
| rs4731925 | C | -0.00829 | 0.00149 | 2.60E-08 |
| rs35169606 | T | 0.008776 | 0.001444 | 1.20E-09 |
| rs11783093 | C | 0.01571 | 0.001897 | 1.20E-16 |
| rs2062882 | G | -0.00811 | 0.00142 | 1.10E-08 |
| rs72674867 | A | 0.008988 | 0.001634 | 3.80E-08 |
| rs4543592 | T | -0.00866 | 0.001389 | 4.50E-10 |
| rs7039819 | G | 0.008734 | 0.001405 | 5.10E-10 |
| rs1246265 | T | -0.00887 | 0.001509 | 4.20E-09 |
| rs1221148 | C | 0.009165 | 0.001407 | 7.30E-11 |
| rs13296519 | G | -0.0097 | 0.001419 | 8.10E-12 |
| rs113382419 | C | -0.02823 | 0.002214 | 3.00E-37 |
| rs11255908 | T | -0.01007 | 0.001588 | 2.30E-10 |
| rs2675638 | G | 0.008499 | 0.0014 | 1.30E-09 |
| rs10823968 | A | 0.008135 | 0.001453 | 2.10E-08 |
| rs17553262 | A | -0.01273 | 0.002181 | 5.30E-09 |
| rs7077678 | C | 0.00855 | 0.001436 | 2.60E-09 |
| rs12244388 | G | -0.01325 | 0.001464 | 1.40E-19 |
| rs3896224 | A | 0.009627 | 0.001418 | 1.10E-11 |
| rs34866095 | A | -0.00857 | 0.001506 | 1.20E-08 |
| rs75742406 | G | 0.009619 | 0.001585 | 1.30E-09 |
| rs17309874 | G | -0.01129 | 0.001582 | 9.70E-13 |
| rs4391802 | A | 0.010319 | 0.001528 | 1.40E-11 |
| rs112282219 | G | -0.02317 | 0.003504 | 3.80E-11 |
| rs9919670 | G | -0.01524 | 0.001421 | 7.60E-27 |
| rs74086911 | G | 0.014802 | 0.002642 | 2.10E-08 |
| rs7297175 | T | -0.00812 | 0.001399 | 6.60E-09 |
| rs10879871 | T | -0.00958 | 0.001458 | 5.00E-11 |
| rs12831617 | C | -0.00918 | 0.001633 | 1.90E-08 |
| rs6562474 | C | 0.008369 | 0.001461 | 1.00E-08 |
| rs7333559 | G | 0.01074 | 0.001707 | 3.20E-10 |
| rs860326 | C | 0.008338 | 0.001402 | 2.70E-09 |
| rs7155595 | A | -0.00885 | 0.001485 | 2.50E-09 |
| rs3742365 | T | -0.01079 | 0.001416 | 2.50E-14 |
| rs35175834 | G | -0.0164 | 0.001698 | 4.60E-22 |
| rs28485305 | C | 0.008007 | 0.001439 | 2.60E-08 |
| rs8042849 | C | 0.019216 | 0.001462 | 1.80E-39 |
| rs8042134 | T | -0.00994 | 0.001401 | 1.30E-12 |
| rs6598539 | T | -0.00815 | 0.001389 | 4.50E-09 |
| rs11861214 | G | 0.009451 | 0.001685 | 2.00E-08 |
| rs12708665 | A | -0.00909 | 0.001539 | 3.50E-09 |
| rs57611503 | G | 0.007743 | 0.00141 | 4.00E-08 |
| rs889398 | C | 0.009241 | 0.001414 | 6.30E-11 |
| rs60952428 | T | 0.013411 | 0.002419 | 3.00E-08 |
| rs1050847 | C | 0.00797 | 0.001405 | 1.40E-08 |
| rs369230 | G | -0.00909 | 0.001511 | 1.80E-09 |
| rs8614 | C | -0.01146 | 0.001797 | 1.80E-10 |
| rs732083 | G | 0.008348 | 0.001473 | 1.50E-08 |
| rs9904288 | T | 0.008428 | 0.001523 | 3.10E-08 |
| rs67596067 | G | -0.00888 | 0.001458 | 1.20E-09 |
| rs12967855 | A | 0.008189 | 0.001479 | 3.10E-08 |
| rs62098013 | G | -0.00857 | 0.001457 | 4.10E-09 |
| rs71367545 | G | -0.01032 | 0.001704 | 1.40E-09 |
| rs76608582 | C | 0.021632 | 0.00344 | 3.20E-10 |
| rs35343344 | C | 0.009181 | 0.001596 | 8.80E-09 |
| rs4814873 | C | 0.009712 | 0.001636 | 2.90E-09 |
| rs6119897 | G | -0.0128 | 0.001627 | 3.60E-15 |
| rs12481282 | G | -0.00894 | 0.001549 | 7.80E-09 |
| rs348809 | A | -0.00828 | 0.001456 | 1.30E-08 |
| rs6011779 | C | 0.019115 | 0.001764 | 2.30E-27 |
| rs147412694 | G | -0.01157 | 0.001949 | 2.90E-09 |
| rs2838834 | C | -0.00936 | 0.001515 | 6.30E-10 |
| rs136233 | A | -0.00996 | 0.001769 | 1.80E-08 |
| rs202645 | A | -0.01016 | 0.001725 | 3.90E-09 |

# **Supplementary Table 7. Genetic variants that were used as proxies for low-density lipoprotein cholesterol (after clumping).**

| Variant | Effect Allele | Effect | Standard Error | P |
| --- | --- | --- | --- | --- |
| rs2419604 | A | 0.0302 | 0.004 | 7.49E-14 |
| rs646776 | T | 0.1602 | 0.0044 | 1.63E-272 |
| rs10893499 | A | 0.0521 | 0.0053 | 3.86E-21 |
| rs10832962 | T | 0.032 | 0.004 | 6.62E-14 |
| rs267733 | A | 0.0331 | 0.0053 | 5.29E-09 |
| rs174583 | C | 0.0522 | 0.0038 | 7.00E-41 |
| rs3184504 | C | 0.0268 | 0.0038 | 4.20E-12 |
| rs1169288 | C | 0.0375 | 0.004 | 6.45E-21 |
| rs2642438 | G | 0.0352 | 0.0042 | 7.32E-16 |
| rs2587534 | A | 0.0391 | 0.0037 | 8.06E-25 |
| rs10903129 | G | 0.0328 | 0.0037 | 3.03E-17 |
| rs12748152 | T | 0.0499 | 0.0066 | 3.21E-12 |
| rs4942486 | T | 0.0243 | 0.0037 | 2.26E-11 |
| rs8017377 | A | 0.0303 | 0.0038 | 2.52E-15 |
| rs2495495 | T | 0.0342 | 0.0059 | 3.52E-08 |
| rs11591147 | G | 0.497 | 0.018 | 8.58E-143 |
| rs7551981 | T | 0.0472 | 0.0038 | 1.36E-33 |
| rs12066643 | C | 0.0389 | 0.0064 | 1.06E-08 |
| rs7534572 | G | 0.0407 | 0.0058 | 1.29E-11 |
| rs247616 | C | 0.0547 | 0.0041 | 2.57E-37 |
| rs2000999 | A | 0.065 | 0.0046 | 4.22E-41 |
| rs6504872 | T | 0.0274 | 0.0037 | 3.48E-13 |
| rs1801689 | C | 0.1028 | 0.0139 | 9.81E-12 |
| rs2886232 | T | 0.0451 | 0.0064 | 3.88E-11 |
| rs314253 | T | 0.0242 | 0.0038 | 3.44E-10 |
| rs6511720 | G | 0.2209 | 0.0061 | 3.85E-262 |
| rs2738459 | A | 0.0532 | 0.0058 | 2.26E-19 |
| rs2228603 | C | 0.104 | 0.0072 | 4.43E-44 |
| rs4970712 | C | 0.0339 | 0.0044 | 2.46E-13 |
| rs2965157 | T | 0.1886 | 0.0112 | 7.29E-62 |
| rs7254892 | G | 0.4853 | 0.0119 | <1E-300 |
| rs75687619 | T | 0.1735 | 0.0161 | 8.05E-24 |
| rs12721109 | G | 0.4462 | 0.0183 | 2.99E-122 |
| rs676388 | C | 0.0265 | 0.0039 | 1.31E-11 |
| rs364585 | G | 0.0249 | 0.0038 | 4.28E-10 |
| rs2328223 | C | 0.0299 | 0.005 | 5.63E-09 |
| rs6016373 | A | 0.0349 | 0.0037 | 7.95E-19 |
| rs6065311 | C | 0.0417 | 0.0036 | 1.66E-30 |
| rs1800961 | C | 0.0685 | 0.0106 | 6.03E-10 |
| rs10490626 | G | 0.0508 | 0.0069 | 1.70E-12 |
| rs2030746 | T | 0.0214 | 0.0038 | 8.61E-09 |
| rs16831243 | T | 0.0378 | 0.0055 | 9.06E-12 |
| rs10195252 | T | 0.0238 | 0.0039 | 3.81E-08 |
| rs1367117 | A | 0.1186 | 0.004 | 9.48E-183 |
| rs72902576 | T | 0.0933 | 0.0133 | 9.58E-12 |
| rs1250229 | C | 0.0243 | 0.0042 | 3.13E-08 |
| rs5763662 | T | 0.0767 | 0.0121 | 1.19E-08 |
| rs11563251 | T | 0.0345 | 0.0062 | 4.50E-08 |
| rs4253776 | G | 0.0311 | 0.0059 | 3.35E-08 |
| rs6544713 | T | 0.0806 | 0.0041 | 4.84E-83 |
| rs6709904 | A | 0.055 | 0.0085 | 4.58E-10 |
| rs2710642 | A | 0.0239 | 0.0038 | 6.09E-09 |
| rs9875338 | G | 0.027 | 0.0037 | 2.21E-11 |
| rs17404153 | G | 0.0336 | 0.0054 | 1.83E-09 |
| rs7640978 | C | 0.0392 | 0.0069 | 9.84E-09 |
| rs6818397 | T | 0.0224 | 0.004 | 1.68E-08 |
| rs4530754 | A | 0.0275 | 0.0036 | 3.58E-12 |
| rs6882076 | C | 0.0456 | 0.0038 | 3.31E-31 |
| rs12916 | C | 0.0733 | 0.0038 | 7.79E-78 |
| rs6909746 | C | 0.0263 | 0.0037 | 7.86E-11 |
| rs112201728 | T | 0.0675 | 0.0104 | 8.51E-10 |
| rs1564348 | C | 0.0481 | 0.005 | 2.76E-21 |
| rs16891156 | C | 0.0965 | 0.0171 | 8.23E-09 |
| rs2315065 | A | 0.1102 | 0.0158 | 5.23E-12 |
| rs3757354 | C | 0.0382 | 0.0044 | 2.09E-17 |
| rs13206249 | G | 0.0378 | 0.0062 | 4.53E-08 |
| rs1408272 | T | 0.052 | 0.0083 | 3.68E-09 |
| rs10947332 | A | 0.0504 | 0.0056 | 6.97E-18 |
| rs2390536 | A | 0.0223 | 0.0038 | 2.04E-08 |
| rs4722551 | C | 0.0391 | 0.0049 | 3.95E-14 |
| rs2073547 | G | 0.0485 | 0.0049 | 1.92E-21 |
| rs2737252 | G | 0.0314 | 0.0041 | 7.04E-14 |
| rs2954029 | A | 0.0564 | 0.0036 | 2.10E-50 |
| rs7832643 | T | 0.0339 | 0.0038 | 2.67E-17 |
| rs13277801 | C | 0.0338 | 0.0038 | 3.99E-17 |
| rs9987289 | G | 0.0714 | 0.0066 | 8.53E-24 |
| rs1883025 | C | 0.0296 | 0.0044 | 6.14E-11 |
| rs579459 | C | 0.0665 | 0.0045 | 2.42E-44 |
| rs3780181 | A | 0.0445 | 0.0074 | 1.76E-09 |
| rs964184 | G | 0.0855 | 0.0078 | 2.01E-26 |

# **Supplementary Table 8. Genetic variants that were used as proxies for systolic blood pressure (after clumping).**

| Variant | Effect allele | Effect | Standard Error | P |
| --- | --- | --- | --- | --- |
| rs1262894 | A | 0.025339 | 0.004476 | 3.30E-08 |
| rs4648815 | G | 0.015551 | 0.002392 | 6.00E-11 |
| rs2493296 | C | -0.02218 | 0.003439 | 1.00E-10 |
| rs12567136 | C | 0.041161 | 0.003184 | 9.60E-38 |
| rs2764834 | T | -0.01686 | 0.002883 | 9.00E-09 |
| rs2782652 | T | -0.0151 | 0.002374 | 4.30E-10 |
| rs1757915 | G | -0.01792 | 0.002466 | 3.00E-13 |
| rs7553325 | C | -0.01347 | 0.002369 | 5.40E-09 |
| rs12035750 | T | -0.01873 | 0.002432 | 4.50E-15 |
| rs3790604 | C | -0.03987 | 0.004527 | 4.40E-18 |
| rs145339349 | G | -0.06034 | 0.008972 | 2.60E-11 |
| rs35479618 | G | -0.06791 | 0.008993 | 2.20E-13 |
| rs861585 | G | -0.01383 | 0.002384 | 8.60E-09 |
| rs7532726 | A | 0.015582 | 0.002676 | 3.00E-09 |
| rs3827750 | C | -0.02859 | 0.003772 | 1.10E-12 |
| rs2867114 | C | 0.022278 | 0.004062 | 1.60E-08 |
| rs4666493 | G | -0.01915 | 0.002398 | 3.30E-15 |
| rs116734066 | C | 0.023368 | 0.004092 | 2.70E-09 |
| rs35021474 | C | 0.025137 | 0.002436 | 2.90E-24 |
| rs72792829 | C | 0.019261 | 0.002861 | 1.10E-11 |
| rs115262049 | A | 0.026098 | 0.004177 | 6.00E-10 |
| rs6708660 | T | 0.014836 | 0.002417 | 3.60E-10 |
| rs6704991 | A | -0.01472 | 0.002469 | 3.20E-09 |
| rs2249105 | A | 0.018909 | 0.002453 | 3.00E-14 |
| rs62162674 | G | 0.014406 | 0.002383 | 3.00E-09 |
| rs10195405 | A | 0.015621 | 0.002602 | 1.30E-09 |
| rs1530558 | T | 0.019562 | 0.003443 | 1.50E-08 |
| rs268263 | T | -0.03043 | 0.00277 | 5.60E-28 |
| rs4246616 | C | -0.01455 | 0.002383 | 3.40E-09 |
| rs1263146 | G | 0.013782 | 0.002375 | 1.10E-08 |
| rs1250258 | C | 0.016996 | 0.002685 | 1.70E-10 |
| rs79349366 | C | 0.042544 | 0.006722 | 6.70E-11 |
| rs2606738 | T | -0.01728 | 0.002517 | 1.30E-12 |
| rs34991912 | T | 0.014303 | 0.002403 | 5.50E-09 |
| rs2643826 | C | -0.01956 | 0.002382 | 3.50E-15 |
| rs6775384 | G | -0.016 | 0.002363 | 1.10E-12 |
| rs4141663 | C | 0.01476 | 0.002405 | 1.10E-09 |
| rs11719057 | G | 0.014533 | 0.002498 | 5.10E-09 |
| rs6787069 | C | -0.01626 | 0.002654 | 3.30E-10 |
| rs11721038 | T | 0.026652 | 0.004501 | 8.90E-09 |
| rs1290784 | C | -0.01986 | 0.00239 | 1.50E-15 |
| rs263017 | A | 0.012817 | 0.002378 | 2.80E-08 |
| rs231708 | G | 0.014433 | 0.002548 | 1.80E-09 |
| rs2610989 | T | -0.01809 | 0.002692 | 5.10E-11 |
| rs55924432 | C | -0.01483 | 0.002416 | 2.00E-09 |
| rs9993149 | T | 0.014974 | 0.002365 | 1.10E-09 |
| rs1051447 | C | 0.014538 | 0.002618 | 4.90E-08 |
| rs7439366 | T | -0.01331 | 0.002372 | 1.50E-08 |
| rs11099098 | G | -0.03856 | 0.002601 | 3.20E-49 |
| rs17011002 | C | -0.02687 | 0.003414 | 5.70E-15 |
| rs10024506 | G | 0.016257 | 0.002788 | 6.80E-09 |
| rs1229984 | T | -0.05775 | 0.007941 | 9.20E-13 |
| rs13107325 | C | 0.033466 | 0.004505 | 3.40E-13 |
| rs6815273 | G | 0.015259 | 0.002411 | 2.20E-09 |
| rs2897515 | T | 0.014075 | 0.002383 | 6.40E-09 |
| rs4690775 | A | 0.014076 | 0.002416 | 3.80E-08 |
| rs12509892 | T | -0.01986 | 0.00237 | 1.50E-16 |
| rs12643599 | A | 0.016923 | 0.002459 | 5.90E-12 |
| rs13129779 | C | 0.013633 | 0.00239 | 1.60E-08 |
| rs12656497 | T | -0.03484 | 0.002409 | 7.10E-46 |
| rs62368019 | T | -0.01527 | 0.002609 | 4.10E-08 |
| rs35807464 | G | -0.02837 | 0.00476 | 1.80E-09 |
| rs6453400 | G | 0.013467 | 0.002385 | 3.60E-08 |
| rs75271047 | G | 0.025114 | 0.004282 | 1.10E-08 |
| rs1422279 | C | -0.0168 | 0.00244 | 5.30E-12 |
| rs17677603 | A | -0.01781 | 0.00243 | 1.50E-13 |
| rs13436194 | A | 0.019425 | 0.002389 | 2.40E-16 |
| rs12055028 | G | -0.01686 | 0.002564 | 1.10E-11 |
| rs72812818 | G | 0.014498 | 0.002578 | 9.40E-09 |
| rs3887266 | C | -0.02079 | 0.003715 | 4.40E-09 |
| rs2607015 | G | -0.02042 | 0.002401 | 4.00E-17 |
| rs9472040 | A | -0.01498 | 0.002532 | 1.40E-09 |
| rs1542977 | G | -0.01502 | 0.002371 | 1.40E-10 |
| rs2499801 | G | 0.019281 | 0.003045 | 7.70E-11 |
| rs13219548 | C | -0.02336 | 0.002382 | 1.40E-22 |
| rs9373523 | T | 0.013685 | 0.002411 | 7.00E-09 |
| rs57139556 | A | 0.03118 | 0.004586 | 8.80E-13 |
| rs434578 | C | 0.020399 | 0.003396 | 1.60E-09 |
| rs9294987 | T | -0.01375 | 0.002383 | 1.90E-08 |
| rs73050466 | G | 0.043543 | 0.007045 | 1.70E-09 |
| rs12699561 | G | -0.01329 | 0.002444 | 1.40E-08 |
| rs57301765 | G | -0.02747 | 0.003257 | 1.80E-16 |
| rs2237306 | C | 0.019843 | 0.003436 | 2.10E-08 |
| rs6461992 | A | -0.03839 | 0.004566 | 9.90E-15 |
| rs1543270 | C | 0.019234 | 0.00238 | 7.40E-15 |
| rs6963105 | A | -0.01481 | 0.002385 | 3.00E-10 |
| rs42038 | C | 0.020199 | 0.002589 | 9.10E-15 |
| rs2392929 | T | -0.04134 | 0.002967 | 1.40E-43 |
| rs2129561 | A | -0.01796 | 0.002415 | 5.00E-13 |
| rs7459106 | A | 0.019134 | 0.003006 | 8.10E-10 |
| rs891511 | G | 0.021302 | 0.002564 | 6.70E-17 |
| rs17173238 | A | -0.01562 | 0.002616 | 5.50E-09 |
| rs13257887 | T | 0.016386 | 0.002713 | 3.30E-09 |
| rs35726503 | A | 0.018517 | 0.002395 | 1.50E-14 |
| rs73563812 | G | 0.021126 | 0.002787 | 1.10E-13 |
| rs2978456 | T | -0.01347 | 0.002374 | 6.50E-09 |
| rs12543884 | A | -0.01833 | 0.0031 | 2.20E-09 |
| rs12544711 | G | 0.015762 | 0.002405 | 7.50E-11 |
| rs2613203 | A | -0.01937 | 0.003046 | 6.60E-10 |
| rs11774829 | T | 0.021893 | 0.003955 | 2.40E-08 |
| rs2469997 | G | 0.020386 | 0.003049 | 4.90E-11 |
| rs9773022 | T | -0.01572 | 0.002374 | 3.30E-11 |
| rs10977670 | A | 0.016119 | 0.00248 | 1.30E-10 |
| rs7019061 | C | 0.014958 | 0.002621 | 1.40E-08 |
| rs10817007 | T | -0.02341 | 0.00354 | 1.30E-10 |
| rs11795365 | G | -0.0195 | 0.003435 | 6.40E-09 |
| rs11145807 | A | 0.014635 | 0.002423 | 2.10E-08 |
| rs1779240 | G | 0.019559 | 0.00279 | 1.10E-12 |
| rs12258967 | C | 0.032925 | 0.00259 | 4.30E-36 |
| rs7070797 | G | 0.032083 | 0.003401 | 1.10E-20 |
| rs10995311 | C | 0.018416 | 0.00239 | 1.20E-14 |
| rs2177843 | C | -0.0224 | 0.003363 | 7.10E-11 |
| rs11187838 | G | 0.024948 | 0.002387 | 1.10E-24 |
| rs10883543 | G | -0.03261 | 0.003772 | 1.60E-16 |
| rs11191580 | T | 0.051648 | 0.004439 | 2.10E-30 |
| rs2782980 | T | -0.01999 | 0.002634 | 8.70E-14 |
| rs11146456 | A | -0.02265 | 0.003723 | 2.50E-09 |
| rs7938342 | T | -0.0289 | 0.002404 | 8.50E-34 |
| rs72843959 | C | 0.014926 | 0.002524 | 2.60E-08 |
| rs56352102 | C | -0.02823 | 0.003054 | 5.20E-20 |
| rs2052692 | A | 0.01431 | 0.002545 | 1.60E-08 |
| rs11821781 | A | -0.01816 | 0.002716 | 3.30E-10 |
| rs11605215 | G | -0.01709 | 0.002994 | 1.30E-08 |
| rs7107356 | A | -0.02338 | 0.002362 | 9.90E-22 |
| rs7125196 | T | 0.02402 | 0.003709 | 4.30E-10 |
| rs2306363 | G | 0.021734 | 0.002922 | 6.80E-14 |
| rs604723 | T | -0.03369 | 0.002661 | 3.10E-37 |
| rs7123754 | T | 0.017661 | 0.002525 | 4.40E-13 |
| rs4937515 | G | 0.018497 | 0.002427 | 8.60E-14 |
| rs1010064 | A | 0.021451 | 0.003059 | 1.10E-12 |
| rs73075659 | A | 0.018087 | 0.002499 | 7.10E-13 |
| rs150857355 | G | -0.04699 | 0.008196 | 4.30E-09 |
| rs113605702 | G | 0.02388 | 0.00427 | 3.00E-09 |
| rs7302981 | A | 0.018192 | 0.002446 | 1.90E-13 |
| rs7315688 | G | 0.014419 | 0.00259 | 3.00E-08 |
| rs7306710 | T | -0.01477 | 0.002384 | 3.80E-09 |
| rs7963801 | T | -0.01339 | 0.0024 | 3.20E-08 |
| rs2681492 | T | 0.03834 | 0.003151 | 8.00E-33 |
| rs597808 | A | 0.02155 | 0.002374 | 1.70E-19 |
| rs35443 | G | 0.020861 | 0.00243 | 1.10E-17 |
| rs12822344 | C | -0.01549 | 0.002399 | 3.30E-10 |
| rs11616710 | C | -0.02707 | 0.004164 | 7.80E-10 |
| rs72683923 | T | 0.053873 | 0.008502 | 5.80E-10 |
| rs12147852 | G | -0.01764 | 0.002762 | 6.00E-11 |
| rs151157965 | G | -0.02446 | 0.004023 | 6.20E-09 |
| rs75989961 | T | -0.026 | 0.004318 | 6.30E-10 |
| rs113044050 | C | 0.019472 | 0.003432 | 2.70E-08 |
| rs11070245 | T | -0.01498 | 0.002377 | 2.80E-10 |
| rs3101442 | T | -0.01632 | 0.002369 | 3.00E-12 |
| rs10775143 | C | 0.013845 | 0.002427 | 8.70E-09 |
| rs2469133 | G | -0.0142 | 0.002494 | 2.40E-08 |
| rs1543927 | T | 0.023704 | 0.002689 | 3.50E-19 |
| rs2062316 | A | -0.01884 | 0.002382 | 1.10E-14 |
| rs8027450 | C | -0.02902 | 0.002536 | 1.20E-30 |
| rs12906962 | T | -0.01483 | 0.002556 | 2.20E-08 |
| rs3211995 | G | 0.023186 | 0.003249 | 3.40E-13 |
| rs2285815 | C | 0.015807 | 0.002675 | 7.00E-09 |
| rs113523959 | G | 0.020622 | 0.003356 | 8.20E-09 |
| rs2303083 | G | 0.025122 | 0.00299 | 1.00E-17 |
| rs7200432 | G | 0.017943 | 0.002589 | 5.80E-12 |
| rs77870048 | C | -0.05013 | 0.005296 | 1.60E-20 |
| rs11641308 | T | -0.01752 | 0.002515 | 1.60E-12 |
| rs4480845 | T | 0.021508 | 0.002479 | 2.80E-18 |
| rs149932962 | G | -0.01619 | 0.002734 | 4.00E-09 |
| rs2111834 | G | 0.02345 | 0.003665 | 1.90E-10 |
| rs2301597 | T | 0.023212 | 0.002401 | 2.30E-21 |
| rs11874 | G | -0.03347 | 0.003438 | 1.00E-19 |
| rs2288276 | G | -0.02196 | 0.003903 | 3.90E-08 |
| rs9907379 | T | -0.02156 | 0.002904 | 1.10E-13 |
| rs3826537 | A | -0.01537 | 0.002391 | 6.20E-11 |
| rs940743 | T | -0.01436 | 0.002376 | 4.70E-09 |
| rs1436138 | A | 0.016155 | 0.002477 | 4.50E-11 |
| rs8076588 | C | 0.015211 | 0.002369 | 4.10E-10 |
| rs1397985 | G | 0.016585 | 0.002532 | 2.40E-10 |
| rs61148001 | C | 0.017464 | 0.002921 | 5.20E-09 |
| rs10048404 | C | 0.014362 | 0.002453 | 4.40E-09 |
| rs12608469 | T | -0.02995 | 0.005008 | 4.40E-09 |
| rs2017199 | G | 0.017527 | 0.002595 | 2.80E-11 |
| rs167479 | G | 0.028235 | 0.002369 | 3.20E-31 |
| rs1077795 | A | 0.015734 | 0.002691 | 6.70E-09 |
| rs3786516 | T | -0.01719 | 0.002883 | 1.50E-08 |
| rs11667829 | G | -0.01411 | 0.002527 | 2.30E-08 |
| rs73046792 | G | 0.022707 | 0.003184 | 6.60E-13 |
| rs11697820 | C | 0.013378 | 0.002414 | 3.00E-08 |
| rs2423514 | A | 0.016346 | 0.002371 | 9.40E-13 |
| rs1887320 | G | -0.01858 | 0.002375 | 3.90E-14 |
| rs75777337 | T | -0.03227 | 0.003875 | 9.90E-17 |
| rs6062625 | T | -0.01759 | 0.00274 | 5.30E-11 |
| rs62229372 | C | -0.02123 | 0.003596 | 2.10E-09 |
| rs137923903 | C | 0.069128 | 0.010372 | 4.80E-10 |
| rs71313932 | G | -0.01513 | 0.002631 | 4.00E-09 |
| rs10212058 | A | 0.017551 | 0.002986 | 4.50E-08 |

# **Supplementary Table 9. Genetic variants that were used as proxies for liability to type 2 diabetes mellitus (after clumping).**

| SNP | Effect Allele | Effect | Standard Error | P |
| --- | --- | --- | --- | --- |
| rs1127215 | T | -0.0470 | 0.0064 | 2.30E-13 |
| rs1493694 | T | 0.0840 | 0.0100 | 2.10E-16 |
| rs145904381 | T | 0.1700 | 0.0310 | 2.20E-08 |
| rs539515 | A | -0.0510 | 0.0080 | 1.20E-10 |
| rs7538328 | T | -0.0420 | 0.0073 | 1.30E-08 |
| rs340874 | T | -0.0680 | 0.0064 | 5.60E-26 |
| rs2494196 | A | -0.0550 | 0.0070 | 6.60E-15 |
| rs348330 | A | -0.0510 | 0.0067 | 3.90E-14 |
| rs291365 | A | 0.0390 | 0.0066 | 4.00E-09 |
| rs3768321 | T | 0.0850 | 0.0080 | 1.30E-26 |
| rs58432198 | T | -0.0650 | 0.0100 | 1.80E-10 |
| rs12140153 | T | -0.0640 | 0.0110 | 1.20E-08 |
| rs147572957 | T | 0.2000 | 0.0370 | 2.90E-08 |
| rs114322470 | T | 0.1800 | 0.0260 | 1.60E-12 |
| rs34872471 | T | -0.3100 | 0.0069 | <1E-300 |
| rs11257655 | T | 0.0900 | 0.0076 | 3.70E-32 |
| rs12769661 | A | -0.0440 | 0.0070 | 3.20E-10 |
| rs2812541 | A | -0.0440 | 0.0064 | 6.10E-12 |
| rs703972 | C | -0.0710 | 0.0064 | 2.50E-28 |
| rs10882101 | T | 0.1100 | 0.0064 | 1.60E-62 |
| rs10893830 | T | -0.0540 | 0.0094 | 7.50E-09 |
| rs10750397 | A | 0.0450 | 0.0070 | 2.00E-10 |
| rs67232546 | T | 0.0560 | 0.0080 | 1.40E-12 |
| rs141521721 | A | 0.1200 | 0.0210 | 2.80E-08 |
| rs5213 | T | -0.0710 | 0.0066 | 1.90E-26 |
| rs78896587 | T | 0.0510 | 0.0088 | 6.10E-09 |
| rs4929965 | A | 0.0700 | 0.0067 | 4.80E-25 |
| rs231360 | T | 0.0600 | 0.0066 | 2.90E-19 |
| rs2237895 | A | -0.0930 | 0.0066 | 3.60E-44 |
| rs145678014 | T | -0.1100 | 0.0160 | 1.10E-11 |
| rs952489 | A | -0.0390 | 0.0071 | 4.80E-08 |
| rs1061810 | A | 0.0500 | 0.0070 | 8.50E-13 |
| rs12798028 | T | 0.0370 | 0.0064 | 9.20E-09 |
| rs1783541 | T | 0.0610 | 0.0080 | 1.40E-14 |
| rs77464186 | A | 0.1100 | 0.0088 | 2.30E-33 |
| rs10830963 | C | -0.0990 | 0.0071 | 1.50E-43 |
| rs3020067 | A | 0.0400 | 0.0068 | 3.90E-09 |
| rs1426371 | A | -0.0500 | 0.0073 | 1.10E-11 |
| rs34965774 | A | 0.0540 | 0.0092 | 3.50E-09 |
| rs56348580 | C | -0.0620 | 0.0069 | 3.80E-19 |
| rs7975763 | T | -0.0450 | 0.0079 | 7.30E-09 |
| rs12811407 | A | 0.0490 | 0.0070 | 2.40E-12 |
| rs10771260 | A | -0.0370 | 0.0065 | 1.70E-08 |
| rs10842994 | T | -0.0740 | 0.0081 | 2.50E-20 |
| rs11063018 | T | -0.0530 | 0.0084 | 1.60E-10 |
| rs4238013 | T | -0.0580 | 0.0080 | 3.30E-13 |
| rs76895963 | T | 0.4800 | 0.0270 | 5.30E-70 |
| rs2258238 | A | -0.1100 | 0.0110 | 2.00E-25 |
| rs1796330 | C | -0.0490 | 0.0064 | 3.20E-14 |
| rs2197973 | T | 0.0350 | 0.0063 | 4.40E-08 |
| rs77864822 | A | 0.0730 | 0.0130 | 2.20E-08 |
| rs7987740 | T | 0.0360 | 0.0065 | 4.10E-08 |
| rs34584161 | A | 0.0480 | 0.0075 | 2.90E-10 |
| rs11842871 | T | -0.0420 | 0.0073 | 1.50E-08 |
| rs9563615 | A | 0.0420 | 0.0070 | 3.90E-09 |
| rs1359790 | A | -0.0830 | 0.0071 | 5.70E-31 |
| rs62007683 | T | -0.0370 | 0.0067 | 3.80E-08 |
| rs17122772 | C | -0.0430 | 0.0077 | 2.00E-08 |
| rs17522122 | T | 0.0380 | 0.0064 | 4.00E-09 |
| rs8018574 | A | -0.0410 | 0.0073 | 2.80E-08 |
| rs17836088 | C | 0.0580 | 0.0077 | 9.70E-14 |
| rs8010382 | A | -0.0380 | 0.0066 | 8.10E-09 |
| rs34715063 | T | -0.0760 | 0.0100 | 3.30E-14 |
| rs2277536 | T | -0.0440 | 0.0069 | 3.10E-10 |
| rs2440374 | C | -0.0410 | 0.0074 | 4.30E-08 |
| rs8037894 | C | -0.0470 | 0.0064 | 3.70E-13 |
| rs7178762 | T | -0.0390 | 0.0063 | 7.00E-10 |
| rs1005752 | A | 0.0790 | 0.0070 | 5.70E-29 |
| rs4932265 | T | 0.0650 | 0.0071 | 7.20E-20 |
| rs2890156 | A | 0.0650 | 0.0092 | 1.50E-12 |
| rs6600191 | T | 0.0610 | 0.0085 | 7.00E-13 |
| rs11642430 | C | -0.0420 | 0.0065 | 1.20E-10 |
| rs3751837 | T | 0.0440 | 0.0077 | 1.70E-08 |
| rs1421085 | T | -0.1200 | 0.0064 | 2.40E-78 |
| rs7196842 | T | -0.0380 | 0.0064 | 5.30E-09 |
| rs72802342 | A | -0.1300 | 0.0120 | 1.30E-27 |
| rs2925979 | T | 0.0530 | 0.0069 | 2.10E-14 |
| rs12920022 | A | 0.0530 | 0.0090 | 2.90E-09 |
| rs4925109 | A | 0.0480 | 0.0068 | 3.90E-12 |
| rs2189301 | A | -0.0600 | 0.0097 | 6.50E-10 |
| rs10908278 | A | -0.0740 | 0.0064 | 3.10E-30 |
| rs1377807 | C | 0.0570 | 0.0068 | 5.70E-17 |
| rs1968866 | T | -0.0770 | 0.0130 | 7.60E-10 |
| rs35895680 | A | -0.0550 | 0.0069 | 3.80E-15 |
| rs60276348 | T | 0.0520 | 0.0095 | 2.90E-08 |
| rs61676547 | C | 0.0550 | 0.0081 | 1.00E-11 |
| rs7222481 | C | 0.0390 | 0.0068 | 1.70E-08 |
| rs62080313 | T | -0.0560 | 0.0098 | 9.10E-09 |
| rs1517037 | T | -0.0460 | 0.0082 | 1.80E-08 |
| rs523288 | A | -0.0560 | 0.0074 | 7.50E-14 |
| rs7240767 | T | -0.0370 | 0.0065 | 2.00E-08 |
| rs3111316 | A | 0.0460 | 0.0065 | 1.60E-12 |
| rs8107974 | A | -0.0930 | 0.0120 | 6.30E-15 |
| rs10406327 | C | 0.0350 | 0.0064 | 4.60E-08 |
| rs429358 | T | 0.0800 | 0.0092 | 1.80E-18 |
| rs10406431 | A | 0.0590 | 0.0065 | 2.50E-19 |
| rs9304665 | A | 0.0450 | 0.0076 | 4.00E-09 |
| rs4804833 | A | 0.0470 | 0.0066 | 1.10E-12 |
| rs11688682 | C | -0.0580 | 0.0075 | 1.40E-14 |
| rs35999103 | T | 0.0520 | 0.0091 | 8.30E-09 |
| rs13426680 | A | 0.0820 | 0.0130 | 6.40E-10 |
| rs1563575 | A | 0.0480 | 0.0072 | 3.80E-11 |
| rs10195252 | T | 0.0600 | 0.0064 | 1.60E-20 |
| rs11680058 | A | 0.0580 | 0.0100 | 1.30E-08 |
| rs2972144 | A | -0.0940 | 0.0066 | 7.90E-46 |
| rs1260326 | T | -0.0670 | 0.0065 | 1.30E-24 |
| rs62107261 | T | 0.1100 | 0.0160 | 1.80E-11 |
| rs80147536 | A | 0.1300 | 0.0110 | 2.70E-30 |
| rs10193538 | T | 0.0370 | 0.0065 | 1.70E-08 |
| rs243024 | A | 0.0580 | 0.0063 | 4.40E-20 |
| rs7608050 | A | -0.0540 | 0.0085 | 1.80E-10 |
| rs12185577 | A | 0.0510 | 0.0065 | 7.20E-15 |
| rs13041756 | T | -0.0580 | 0.0100 | 1.30E-08 |
| rs2268078 | A | 0.0430 | 0.0067 | 2.90E-10 |
| rs1800961 | T | 0.1600 | 0.0170 | 3.20E-20 |
| rs1999536 | C | -0.0410 | 0.0064 | 1.30E-10 |
| rs11699802 | T | -0.0430 | 0.0064 | 2.50E-11 |
| rs6070625 | C | -0.0440 | 0.0063 | 3.20E-12 |
| rs6518681 | A | -0.0830 | 0.0120 | 9.60E-13 |
| rs117001013 | T | -0.0650 | 0.0110 | 1.50E-08 |
| rs5758223 | A | 0.0380 | 0.0070 | 4.60E-08 |
| rs1801645 | T | -0.0480 | 0.0074 | 1.50E-10 |
| rs11708067 | A | 0.0890 | 0.0076 | 1.30E-31 |
| rs11709077 | A | -0.1100 | 0.0098 | 1.60E-27 |
| rs4679370 | T | -0.0390 | 0.0064 | 1.40E-09 |
| rs62271373 | A | 0.0880 | 0.0140 | 1.00E-09 |
| rs12630224 | T | 0.0380 | 0.0066 | 1.50E-08 |
| rs7629630 | A | 0.0510 | 0.0091 | 2.20E-08 |
| rs9873618 | A | -0.0660 | 0.0070 | 8.50E-21 |
| rs6780171 | A | 0.1100 | 0.0068 | 2.50E-58 |
| rs9814673 | T | 0.0410 | 0.0070 | 5.90E-09 |
| rs4686471 | T | -0.0600 | 0.0065 | 3.10E-20 |
| rs35352848 | T | 0.0710 | 0.0079 | 9.50E-20 |
| rs4688760 | T | 0.0430 | 0.0069 | 4.50E-10 |
| rs2292662 | T | -0.0650 | 0.0089 | 3.30E-13 |
| rs9860730 | A | 0.0550 | 0.0070 | 7.40E-15 |
| rs2272163 | A | -0.0370 | 0.0065 | 1.20E-08 |
| rs1580278 | A | -0.0410 | 0.0064 | 2.90E-10 |
| rs1296328 | A | 0.0350 | 0.0064 | 4.30E-08 |
| rs7669833 | A | -0.0540 | 0.0070 | 1.80E-14 |
| rs12640250 | A | -0.0390 | 0.0071 | 4.50E-08 |
| rs4865436 | C | -0.0460 | 0.0075 | 1.00E-09 |
| rs58730668 | T | 0.0680 | 0.0092 | 1.00E-13 |
| rs10938398 | A | 0.0440 | 0.0064 | 4.90E-12 |
| rs2102278 | A | -0.0380 | 0.0069 | 4.50E-08 |
| rs10937721 | C | 0.0870 | 0.0065 | 1.60E-40 |
| rs1531583 | T | 0.1100 | 0.0150 | 1.20E-12 |
| rs138641407 | A | 0.0420 | 0.0067 | 5.70E-10 |
| rs6821438 | A | 0.0420 | 0.0063 | 5.40E-11 |
| rs115505614 | T | 0.1700 | 0.0150 | 1.70E-29 |
| rs329122 | A | 0.0370 | 0.0064 | 9.20E-09 |
| rs6885132 | C | 0.0780 | 0.0110 | 9.50E-13 |
| rs11958808 | C | 0.0380 | 0.0065 | 5.80E-09 |
| rs2648731 | A | 0.0460 | 0.0076 | 2.10E-09 |
| rs702634 | A | 0.0510 | 0.0069 | 2.10E-13 |
| rs459193 | A | -0.0730 | 0.0073 | 4.40E-23 |
| rs2307111 | T | 0.0530 | 0.0065 | 3.30E-16 |
| rs4457053 | A | -0.0590 | 0.0069 | 1.40E-17 |
| rs72764969 | A | 0.0400 | 0.0065 | 1.30E-09 |
| rs4946812 | A | -0.0390 | 0.0068 | 1.00E-08 |
| rs11759026 | A | -0.0670 | 0.0075 | 1.30E-18 |
| rs1573090 | T | 0.0500 | 0.0064 | 8.40E-15 |
| rs474513 | A | 0.0390 | 0.0063 | 1.00E-09 |
| rs4709746 | T | -0.0560 | 0.0096 | 5.00E-09 |
| rs7756992 | A | -0.1400 | 0.0070 | 3.00E-87 |
| rs2857605 | T | 0.0610 | 0.0077 | 4.80E-15 |
| rs601945 | A | -0.0800 | 0.0085 | 2.70E-21 |
| rs6458354 | T | -0.0510 | 0.0070 | 3.70E-13 |
| rs3798519 | A | -0.0580 | 0.0082 | 1.10E-12 |
| rs648795 | A | 0.0420 | 0.0065 | 1.00E-10 |
| rs9379084 | A | -0.0970 | 0.0110 | 2.30E-20 |
| rs11496066 | T | 0.0470 | 0.0083 | 1.20E-08 |
| rs6976111 | A | 0.0420 | 0.0073 | 1.50E-08 |
| rs1562396 | A | -0.0580 | 0.0069 | 7.60E-17 |
| rs17168486 | T | 0.0690 | 0.0083 | 6.90E-17 |
| rs1117610 | A | -0.0440 | 0.0076 | 1.10E-08 |
| rs10228066 | T | 0.0660 | 0.0063 | 1.90E-25 |
| rs6459733 | C | -0.0580 | 0.0068 | 3.90E-17 |
| rs1708302 | T | -0.0920 | 0.0063 | 4.20E-48 |
| rs58682124 | A | 0.0440 | 0.0075 | 4.80E-09 |
| rs878521 | A | 0.0570 | 0.0074 | 1.60E-14 |
| rs3802177 | A | -0.1100 | 0.0069 | 6.30E-55 |
| rs17772814 | A | -0.0780 | 0.0130 | 5.00E-10 |
| rs1561927 | T | -0.0430 | 0.0071 | 1.90E-09 |
| rs4977213 | T | -0.0510 | 0.0067 | 4.40E-14 |
| rs12719778 | T | 0.0390 | 0.0064 | 2.10E-09 |
| rs263 | T | -0.0480 | 0.0085 | 1.40E-08 |
| rs10954772 | T | 0.0410 | 0.0068 | 2.30E-09 |
| rs13262861 | A | -0.0940 | 0.0087 | 1.80E-27 |
| rs10097617 | T | 0.0510 | 0.0063 | 1.10E-15 |
| rs17689007 | A | -0.0480 | 0.0064 | 1.70E-13 |
| rs505922 | T | -0.0460 | 0.0067 | 5.40E-12 |
| rs28505901 | A | -0.0760 | 0.0081 | 2.60E-21 |
| rs7022807 | A | -0.0400 | 0.0064 | 3.60E-10 |
| rs2383205 | A | -0.0540 | 0.0065 | 1.30E-16 |
| rs10811660 | A | -0.1600 | 0.0086 | 6.60E-79 |
| rs1412234 | T | -0.0430 | 0.0068 | 2.50E-10 |
| rs12001437 | T | -0.0410 | 0.0065 | 3.70E-10 |
| rs10974438 | A | -0.0510 | 0.0066 | 1.60E-14 |
| rs17791513 | A | 0.1000 | 0.0130 | 2.90E-14 |
| rs2796441 | A | -0.0660 | 0.0065 | 8.50E-24 |
| rs55653563 | A | 0.0430 | 0.0072 | 3.20E-09 |

# **Supplementary Table 10: Pleiotropy robust Mendelian randomization method estimates for analyses investigating the association of genetic liability to type 2 diabetes with lung *ACE2* expression in the GTEx project and Lung eQTL consortium and with plasma concentrations of ACE2 in INTERVAL.**

Estimates (95% confidence intervals; CI) represent the change in the outcome (in standard deviation units) per unit increase in the log_e_ odds of genetic liability to type 2 diabetes mellitus.

|  | **GTEx** | | **Lung eQTL consortium** | | **INTERVAL** | |
| --- | --- | --- | --- | --- | --- | --- |
| **Method** | **Estimate (95% CI)** | **P** | **Estimate (95% CI)** | **P** | **Estimate (95% CI)** | **P** |
| **Inverse-variance weighted** | 0.244 (0.108, 0.379) | 4x10^-4^ | -0.013 (-0.075, 0.049) | 0.68 | 0.057 (0.006, 0.108) | 0.029 |
| **Weighted median** | 0.295 (0.090, 0.500) | 0.01 | -0.011 (-0.135, 0.113) | 0.86 | 0.055 (-0.045,0.154) | 0.28 |
| **MR-Egger** | 0.345 (-0.038, 0.727) | 0.08 | -0.010 (-0.146, 0.126) | 0.89 | 0.028 (-0.083, 0.139) | 0.62 |
| **(intercept)** | -0.007 (-0.031, 0.017) | 0.58 | 0.000 (-0.009, 0.009) | 0.96 | 0.002 (-0.005, 0.010) | 0.56 |
| **Contamination mixture** | 0.286 (0.072, 0.497) | 0.01 | 0.047 (-0.061, 0.130) | 0.38 | 0.132 (0.056, 0.221) | 2x10^-3^ |
